# Spectral-YOLOv13: A Dual-Domain Vision-Mamba Sensing Framework for Fine-Grained Coral Health Assessment and Continuous Ecological Forecasting

**DOI:** 10.3390/s26103265

**Published:** 2026-05-21

**Authors:** Litian Yang, Wenkun Chen, Zhuoyue Mo, Xin Gao, Minzhi Mo, Chunlei Xia, Liankuan Zhang

**Affiliations:** 1 College of Mathematics and Informatics, South China Agricultural University, Guangzhou 510642, China; yanglitian@stu.scau.edu.cn (L.Y.); goxing@stu.scau.edu.cn (X.G.); saber_mo@stu.scau.edu.cn (M.M.); 2 College of Software Engineering, South China Agricultural University, Guangzhou 510642, China; 19129213013@163.com (W.C.); 13922518637@163.com (Z.M.); 3 Yantai Institute of Coastal Zone Research, Chinese Academy of Sciences, Yantai 264003, China; clxia@yic.ac.cn

**Keywords:** precision ecological sensing, underwater vision sensing, intelligent sensing system, real-time object detection, state space models, fine-grained ecological classification, coral health monitoring, underwater robotics, continuous ecological forecasting

## Abstract

Coral reefs are among the most important and vulnerable marine ecosystems worldwide. AI-powered underwater visual monitoring has become essential for effective reef conservation, yet current methods still face severe limitations: spectral ambiguity caused by underwater turbidity, fine-grained confusion in early coral health assessment, and discrete forecasting models that cannot represent continuous ecological degradation dynamics. To address these issues, we propose Spectral-YOLOv13, a dual-domain vision-Mamba sensing framework for high-precision coral health evaluation and continuous ecological forecasting. The framework incorporates three novel components: a Wavelet-Integrated Omni-Neck (WIO-Neck) to perform multi-scale spectral filtering and suppress turbidity-induced noise; a Contrastive Prototype Head (CP-Head) to enhance discriminability between visually similar health states; and a Bio-Mamba Predictor based on state-space models to capture long-term continuous health trajectories. Extensive experiments on the CR-Mix++ dataset demonstrate that Spectral-YOLOv13 achieves 53.8% mAP with strong robustness in turbid underwater environments. It reduces four-week forecasting error by 26.8% and maintains real-time inference speed at 112 FPS. This work provides a reliable and high-performance vision framework for practical underwater coral reef monitoring and proactive conservation management.

## 1. Introduction

Coral reefs are among the most productive and biologically diverse marine eco-systems on Earth, providing critical ecological services such as coastal protection, bio-diversity maintenance, fishery support, and climate regulation [1,2]. However, climate change and increasing anthropogenic pressure have transformed coral bleaching from episodic events into a chronic and accelerating global crisis [3]. Once coral health deteriorates to an irreversible state, ecosystem structure and function can be permanently damaged. Therefore, high-precision, real-time, and long-term coral health monitoring has become an essential task in marine conservation and marine biological research.

From the perspective of marine biology, coral health degradation is a continuous and gradual physiological process, especially in the early stages. Subtle changes such as initial discoloration, reduced polyp expansion, and micro-lesions are critical early-warning signals of bleaching [4]. These fine-grained health transitions—between healthy, sub-healthy, bleached, and dead states—are difficult to identify through manual observation or conventional imaging methods [5]. Misdiagnosis or delayed detection often leads to missed windows for effective intervention. Thus, fine-grained coral health assessment is not only a technical demand but also a key scientific issue in coral reef ecology.

From the perspective of underwater optics, imaging-based coral monitoring faces severe environmental challenges. Underwater environments are characterized by strong light attenuation, wavelength-dependent scattering, suspended particle turbidity, and backscatter noise [6]. These factors cause spectral distortion, contrast loss, and high-frequency noise in captured images, which significantly degrade visual feature quality. Traditional computer vision models that rely solely on spatial-domain convolution lack explicit mechanisms to suppress turbidity-induced interference and often suffer from unstable detection and severe performance degradation in complex underwater optical environments.

In recent years, the integration of deep learning with autonomous underwater vehicles (AUVs) has revolutionized marine monitoring. Object detectors, particularly the YOLO (You Only Look Once) series, have become the de facto standard for this task due to their real-time inference capabilities [7,8]. Significant strides have been made to adapt these general-purpose models to the underwater domain. For instance, the Automatic Coral Detection with YOLO framework provides efficient coral reef detection via deep learning [9]. While such advancements have improved detection accuracy for small and occluded objects, we identify two fundamental limitations that persist in current state-of-the-art methodologies, including the latest YOLOv13-based adaptations.

The first limitation arises from environment-induced spectral degradation in underwater imagery. Turbidity, suspended particles, and wavelength-dependent light attenuation introduce high-frequency noise and contrast loss that substantially degrade visual quality [10]. Most existing detectors operate purely in the spatial domain, relying on convolutional filters to implicitly suppress noise. In practice, this approach becomes unstable under varying water clarity conditions, where stochastic turbidity noise overwhelms biologically meaningful coral structures, leading to pronounced performance degradation. Such instability is widely observed in underwater visual tasks under complex optical conditions, including low-light environments, high suspended sediment, and uneven illumination.

The second limitation lies in the “fine-grained confusion” and the discrete nature of ecological forecasting. Identifying the transitional “Sub-healthy” state—a critical early-warning sign of bleaching—is a fine-grained classification problem. Standard detection heads, which optimize a simple cross-entropy loss, often fail to separate the subtle textural shifts in early bleaching from healthy tissues, leading to high misclassification rates. Furthermore, current temporal forecasting modules typically rely on Recurrent Neural Networks (e.g., ConvLSTM) [11]. These architectures suffer from high computational complexity and a “short-term memory” bias, making them ill-suited for modeling the continuous, long-term non-linear dynamics of biological decay on edge devices.

To address these challenges, we propose Spectral-YOLOv13, a dual-domain vision framework for fine-grained coral health assessment and continuous ecological forecasting. Built upon the YOLOv13 backbone, the proposed system is organized as a three-layer decision-support pipeline encompassing perception, understanding, and prediction.

At the perception layer, we introduce the Wavelet-Integrated Omni-Neck (WIO-Neck), which explicitly incorporates frequency-domain priors into feature aggregation. By decomposing features using the Discrete Wavelet Transform (DWT), WIO-Neck separates structure-dominated low-frequency components from turbidity-induced high-frequency noise, enabling adaptive spectral filtering under heterogeneous water conditions and providing a stable perceptual foundation for downstream analysis. At the understanding layer, we design a Contrastive Prototype Head (CP-Head) to resolve fine-grained ambiguity between adjacent coral health states. CP-Head integrates supervised contrastive learning into the detection head, representing each health category with a learnable prototype and enforcing margin-aware separation in the embedding space. This design improves confidence calibration and decision reliability, particularly at the critical Healthy–Sub-healthy boundary. At the prediction layer, we propose the Bio-Mamba Predictor, a lightweight vision state-space model that treats coral health evolution as a continuous biological process rather than a sequence of discrete transitions. Leveraging the linear complexity and selective scanning mechanism of Mamba [12], this module treats coral health trajectories as continuous signals rather than discrete snapshots, enabling efficient and stable long-horizon forecasting suitable for deployment on AUV platforms with a fraction of the computational cost of Transformers or LSTMs.

In summary, this work makes the following contributions:

1. Robust spectral perception for degraded environments: We introduce WIO-Neck, a wavelet-integrated feature aggregation module that explicitly mitigates turbidity-induced noise, improving detection stability under real-world underwater conditions.
2. Decision-critical fine-grained classification: We propose CP-Head, a contrastive prototype-based detection head that enhances feature separability and confidence calibration for early-stage coral health assessment.
3. Efficient continuous ecological forecasting: We present Bio-Mamba, a state-space-based temporal predictor that models coral degradation as a continuous process, enabling accurate and computationally efficient long-horizon forecasting suitable for edge deployment.
4. Practical decision-support framework: By unifying detection and forecasting within a single architecture, Spectral-YOLOv13 provides a scalable and reliable visual monitoring solution for long-term reef management, directly aligns with the demands of marine ecological monitoring and coral reef conservation applications.

## 2. Related Work

Detecting small targets in complex underwater environments is an increasingly critical area of research, particularly for identifying coral colonies and early signs of bleaching. This section provides a comprehensive overview of recent advancements and methodologies relevant to small-target detection, addressing the challenges presented by existing approaches, including Coral-YOLO, and highlighting areas for further investigation.

### 2.1. Small-Target Detection Techniques

Small-target detection in underwater settings has seen remarkable advancements driven by innovations in machine learning and computer vision. Wang et al. (2025) [13] introduced PC-YOLO11s, a lightweight and effective feature extraction method specifically designed for detecting small targets in challenging conditions, such as murky waters. Their findings demonstrate that lightweight models can achieve high accuracy and efficiency, making them suitable for real-time underwater applications [13].

Complementing this, Liu et al. (2022) developed an integrated underwater image enhancement and biological detection pipeline that effectively improves visibility and detection precision for small marine organisms [14].

Cheng et al. (2024) further improved small-target representation by introducing dynamic convolution and global attention mechanisms, which strengthen the extraction of weak features and suppress background clutter in turbid underwater scenes [15].

### 2.2. Limitations of Typical Underwater Detectors

While deep learning-based detectors have been widely applied in coral monitoring, their performance degrades significantly under complex aquatic conditions. Tao et al. (2025) noted that frameworks such as Coral-YOLO suffer from performance drops in turbid waters, largely due to insufficient modeling of underwater optical distortion [9].

Similarly, real-world underwater detection frequently fails to identify tiny early-bleaching spots, as conventional detection heads lack fine-grained feature discriminability and robustness to noise [16].

Khan et al. (2024) summarized that most existing underwater detectors struggle with scale variance, color distortion, and low contrast, which directly affect the reliability of long-term autonomous monitoring [17].

### 2.3. Advances in Deep Learning for Underwater Image Processing

Recent studies have highlighted the significant role of deep learning in improving the quality and reliability of underwater image processing. Li et al. (2020) established a comprehensive benchmark dataset for underwater image enhancement, providing a standardized framework for evaluating various deep learning approaches [18].

Furthermore, Zhang et al. (2021) [19] developed a GAN-based method for enhancing underwater images. Their approach significantly improved contrast and visibility, which are crucial for detecting small features such as coral bleaching and other underwater anomalies [19].

Saoud and Hussain (2025) proposed EBA-AI, an efficient enhancement framework that reduces computational overhead while improving generalization across diverse underwater visual conditions, supporting reliable deployment in large-scale reef monitoring [20].

### 2.4. Keypoint Identification and Anomaly Detection

Identifying keypoints within underwater images is vital for detecting anomalies or subtle changes, such as those indicative of bleaching. Li et al. (2024) proposed a multi-scale perception and enhancement algorithm for underwater organisms, which effectively strengthens small-object feature extraction and reduces information loss [21].

Additionally, Ferrera et al. (2019) presented a real-time monocular visual odometry system tailored for turbid and dynamic underwater environments, demonstrating the importance of robust detection algorithms that can adapt effectively to these complexities [22].

### 2.5. Conclusion and Future Directions

In summary, existing underwater small-target detection methods still face three core limitations: (1) poor robustness under turbidity, uneven illumination, and spectral distortion; (2) low accuracy in fine-grained and early-warning coral health recognition; (3) the lack of stable, efficient designs suitable for real-world underwater deployment. This work addresses these gaps by developing a robust coral detection framework that integrates multi-scale small-target modeling, spectral denoising, and fine-grained contrastive learning.

## 3. Methodology

### 3.1. The Spectral-YOLOv13 Framework

Unlike existing coral detection systems that focus solely on segmentation or localization, Spectral-YOLOv13 is designed as a complete decision-support system comprising three functionally distinct but synergistic layers built upon a YOLOv13-L backbone. This hierarchical architecture reflects the progression from sensory data to actionable intelligence:

- Layer 1: Perception Layer (WIO-Neck). This solves the “spectral ambiguity” problem by explicitly filtering turbidity-induced high-frequency noise in the wavelet domain, enabling robust feature extraction regardless of water clarity.
- Layer 2: Understanding Layer (CP-Head). This solves the “fine-grained confusion” problem by using contrastive learning to construct discriminative feature prototypes, forcing the network to maximize decision boundaries between visually similar health states—critical for early warning.
- Layer 3: Prediction Layer (Bio-Mamba). This solves the “discrete forecasting” problem by modeling coral degradation as a continuous biological trajectory rather than discrete snapshots, enabling long-term ecosystem forecasting with linear computational complexity.

**Figure 1:**
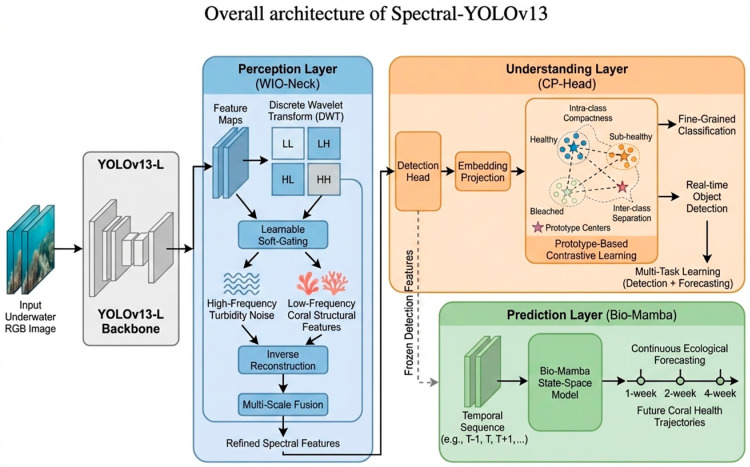
Overall architecture of the proposed Spectral-YOLOv13 framework.

The entire framework is trained end-to-end using a compound loss function that balances localization, classification, contrastive learning, and temporal consistency. The overall architecture of Spectral-YOLOv13 is illustrated in Figure 1, highlighting the hierarchical interaction between perception, understanding, and prediction layers.

The model is organized as a three-layer decision-support pipeline built upon a YOLOv13-L backbone. The perception layer (WIO-Neck) performs wavelet-based spectral filtering to suppress turbidity-induced noise. The understanding layer (CP-Head) introduces prototype-based contrastive learning for fine-grained health classification. The prediction layer (Bio-Mamba) models coral health evolution as a continuous dynamical process using a state-space architecture. Detection and forecasting branches operate jointly through a multi-task learning framework.

### 3.2. Layer 1: Perception Layer-Wavelet-Integrated Omni-Neck (WIO-Neck)

#### 3.2.1. Motivation and Spectral Analysis

Conventional Feature Pyramid Networks (FPNs) implicitly rely on convolutional kernels to suppress high-frequency noise through spatial learning. In highly turbid underwater environments, however, stochastic particulate noise dominates the visual signal, leading to conflicting optimization objectives between noise suppression and structural feature preservation and, consequently, degraded generalization across turbidity levels.

To overcome this limitation, we explicitly impose a spectral prior in which coral structural information is primarily encoded in low-frequency components, while turbidity-induced noise is concentrated in high frequencies. An a priori frequency analysis on the CR-Mix++ dataset supports this assumption: high-frequency sub-bands ($F^{HH}$) exhibit a strong correlation with turbidity (Spearman ρ = 0.82), whereas low-frequency components ($F^{LL}$) remain largely independent (ρ = 0.12). As turbidity increases from clear to high conditions, the relative energy of $F^{HH}$ increases from 4.4% to 12.2%, while that of $F^{LL}$ decreases accordingly, demonstrating that spectral decomposition effectively separates morphological signal from environmental noise.

#### 3.2.2. Explicit Spectral Decomposition and Soft-Gating

We implement this prior by embedding a 2-level Discrete Wavelet Transform (DWT) using the Daubechies (Db4) basis at each feature pyramid level i (from P3 to P7). For an input feature map $F_{i}$, the DWT produces four sub-bands: $F_{i}^{LL}$ (coarse coral morphology as branching skeletons and polyp patterns), $F_{i}^{LH}$ and $F_{i}^{HL}$ (edge and texture gradients), and $F_{i}^{HH}$ (high-frequency stochastic noise as marine snow and backscatter). Instead of using fixed-threshold shrinkage, we introduce a learnable soft-gating mechanism to adaptively modulate these components based on local feature statistics:

(1) $$F_{i}^{LL^{\prime }}=F_{i}^{LL}⊙\left(1+\alpha_{i}\;\sigma \left(W_{LL}*F_{i}^{LL}\right)\right)$$

(2) $$F_{i}^{HH^{\prime }}=F_{i}^{HH}⊙\left(1-\beta_{i}\;\sigma \left(W_{HH}*F_{i}^{HH}\right)\right)$$

(3) $$F_{i}^{LH^{\prime }}=F_{i}^{LH}⊙\left(1+\gamma_{i}\;\sigma \left(W_{LH}*F_{i}^{LH}\right)\right)$$

(4) $$F_{i}^{HL^{\prime }}=F_{i}^{HL}⊙\left(1-\delta_{i}\;\sigma \left(W_{HL}*F_{i}^{HL}\right)\right)$$

**Figure 2:**
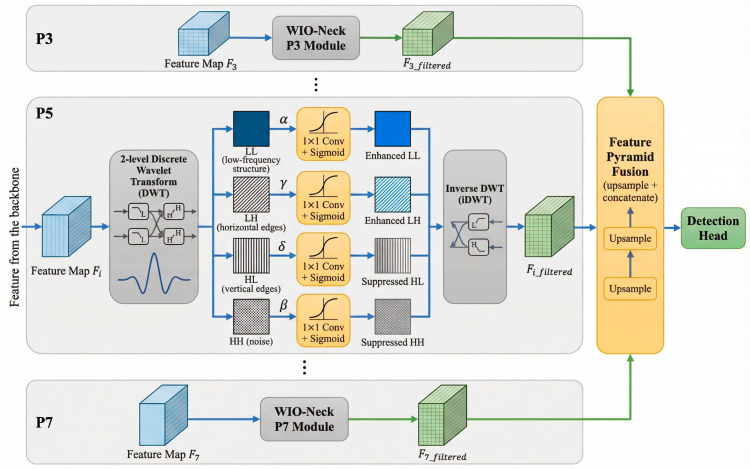
Architecture of the Wavelet-Integrated Omni-Neck (WIO-Neck). Feature maps from different pyramid levels are decomposed using a two-level Discrete Wavelet Transform (DWT), generating LL, LH, HL, and HH sub-bands. Learnable soft-gating mechanisms adaptively enhance structure-dominated components while suppressing noise-dominated bands. Filtered sub-bands are reconstructed via inverse DWT and fused through standard feature pyramid operations.

Here, ⊙ denotes element-wise multiplication, σ is the sigmoid function, $\alpha_{i},\beta_{i},\gamma_{i},\delta_{i}$ are learnable per-level scaling factors, and $W_{*}$ are lightweight 1 × 1 convolutional filters, and ∗ denotes the convolution operation. This formulation selectively enhances structure-dominated components (LL,LH) while suppressing noise-dominated bands (HL,HH), enabling content-aware spectral denoising. The detailed architecture of the proposed WIO-Neck is illustrated in Figure 2.

#### 3.2.3. Reconstruction and Implementation

After gating, an inverse DWT reconstructs the filtered feature map:

(5) $$F_{i}^{filtered}=\;iDWT(F_{i}^{LL^{\prime }},F_{i}^{LH^{\prime }},F_{i}^{HL^{\prime }},F_{i}^{HH^{\prime }})$$

The reconstructed features are then fused across scales using standard FPN operations (upsampling and concatenation), ensuring that every pyramid level benefits from explicit spectral denoising rather than relying solely on the final detection layers.

From a computational perspective, fast wavelet transforms operate in O(N) time. Consequently, WIO-Neck introduces only a ~12% FLOPs increase relative to a standard FPN. Ablation results confirm that the Db4 basis provides the optimal balance between localization and smoothness, and that a 2-level decomposition aligns well with typical coral feature scales (5 mm–2 cm), outperforming both shallower (insufficient denoising) and deeper (loss of fine detail) alternatives.

### 3.3. Layer 2: Understanding Layer-Contrastive Prototype Head (CP-Head)

#### 3.3.1. Motivation and Contrastive Framework

Standard object detection heads typically employ decoupled classification branches optimized using cross-entropy loss. While effective for coarse classification, this objective only encourages the correct class to receive the highest predicted probability and does not explicitly enforce separation or margin between classes in the feature space. As a result, for visually similar categories such as Healthy and Sub-healthy corals, cross-entropy-based classifiers often suffer from severe boundary ambiguity: samples near class boundaries are prone to misclassification, and even correctly detected Sub-healthy instances tend to receive low confidence scores (typically in the range of 0.65–0.72). Moreover, the absence of margin awareness leads to poor confidence calibration, where predicted probabilities fail to reflect the model’s true uncertainty in borderline cases.

To address these limitations, we replace the standard decoupled head with a Contrastive Prototype Head (CP-Head). Each health class is represented by a learnable prototype vector serving as the class center in the embedding space. For each detection sample, the network outputs a feature embedding through the shared backbone–neck–head encoder, and training is guided by a supervised contrastive learning objective that explicitly pulls samples of the same health state closer while pushing samples from different states apart.

(6) $$L_{SupCon}=-\frac{1}{\left|P(i)\right|}\sum_{p\in P(i)}\log\frac{\exp\left(z_{i}\cdot z_{p}/\tau \right)}{\sum_{a=1}^{N}\exp\left(z_{i}\cdot z_{a}/\tau \right)}$$

**Figure 3:**
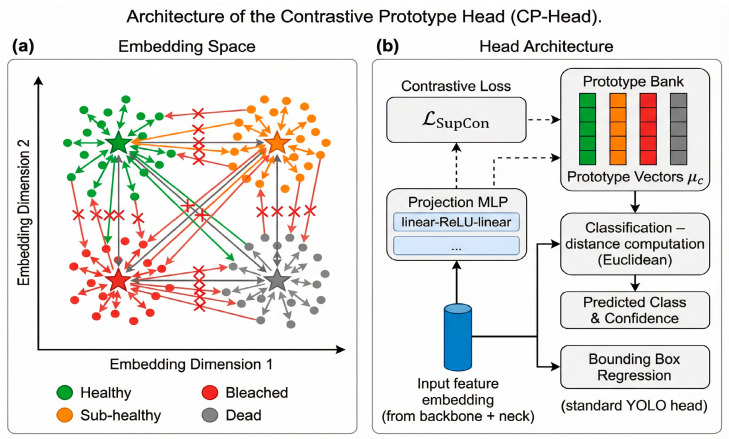
Architecture of the Contrastive Prototype Head (CP-Head). Feature embeddings are projected into a prototype-centered embedding space where each coral health state is represented by a learnable class prototype. Supervised contrastive learning encourages intra-class compactness and inter-class separation, improving decision boundary clarity for fine-grained classification.

From a biological perspective, this formulation encourages healthy corals to cluster around features reflecting intact morphology, sub-healthy corals to activate distinctive early stress patterns, and severely degraded corals to occupy clearly separated regions corresponding to tissue damage and bleaching. The architecture of the Contrastive Prototype Head (CP-Head) is illustrated in Figure 3.

#### 3.3.2. Prototype Maintenance and Joint Loss Function

During training, we maintain a dynamic prototype bank for all health classes. Prototypes are initialized using class-wise feature averages and updated via a momentum-based scheme to ensure stability while allowing gradual adaptation to evolving feature distributions.

(7) $$\mu_{c}^{\left(t+1\right)}=(1-m)\mu_{c}^{\left(t\right)}+m\cdot mean(\{f_{\phi }(x_{i}):y_{i}=c\})$$

At inference time, classification is performed by assigning each detection to the nearest class prototype in the normalized embedding space.

(8) $$\hat{c}=\arg\underset{c}{\min}||norm(z)-norm(\mu_{c})|{|}_{2}$$

Prediction confidence is derived from the relative distance to the decision boundary defined by competing prototypes, providing an interpretable measure of uncertainty that is directly tied to feature separability rather than softmax probabilities. The final detection objective combines localization loss, classification loss, and supervised contrastive loss with empirically selected weights.

Specifically, $L_{Loc}$ refers to the CIoU loss for bounding box regression, which is the default and standard localization loss function in YOLOv13.

$L_{CE}$ denotes the cross-entropy classification loss, and $L_{SupCon}$ denotes the supervised contrastive loss.

(9) $$L_{detection}=\lambda_{1}L_{Loc}+\lambda_{2}L_{CE}+\lambda_{3}L_{SupCon}$$

The specific weights used in our final configuration are $\lambda_{1}$ = 1.0, $\lambda_{2}$ = 0.3, and $\lambda_{3}$ = 0.7, as determined through systematic loss-weight optimization (see Section 4.5.3). In this formulation, the contrastive component primarily shapes the feature space structure, while the cross-entropy term acts as a regularizer to stabilize optimization. This joint design yields more robust convergence than using contrastive learning alone and is particularly effective for fine-grained health state discrimination.

#### 3.3.3. Feature Space Structure Induced by Contrastive Prototypes

Standard cross-entropy-based detection heads optimize class likelihoods without explicitly constraining feature geometry, which often results in ambiguous decision boundaries for visually similar categories. This limitation is particularly pronounced in fine-grained coral health assessment, where class imbalance and subtle visual differences can lead to clustered misclassifications near class boundaries and poorly calibrated confidence scores.

In contrast, the proposed CP-Head introduces prototype-centered supervision that explicitly enforces inter-class separation in the embedding space. By jointly optimizing cross-entropy and supervised contrastive objectives, CP-Head encourages compact intra-class clustering while enlarging margins between adjacent health states, leading to more discriminative and better-calibrated representations.

To analyze the resulting feature structure, we visualize detection embeddings using t-SNE. Compared with the YOLOv13 baseline and a standard supervised contrastive variant, CP-Head yields tighter intra-class clusters and clearer separation between health states, most notably along the Healthy–Sub-healthy boundary. Quantitative clustering metrics further confirm this behavior, showing consistently larger inter-class margins and higher separation scores than competing methods.

### 3.4. Layer 3: Predictive Layer-Bio-Mamba Predictor

#### 3.4.1. Problem Statement: Discrete vs. Continuous Ecological Forecasting

Most existing temporal forecasting approaches in coral monitoring model reef health evolution as a sequence of discrete states, typically implemented using recurrent architectures such as ConvLSTM:

(10) $$h_{t}=LSTM(x_{t},h_{t-1})$$

While effective for short-term prediction, this discrete formulation is suboptimal for long-horizon forecasting. Recurrent models suffer from vanishing gradients when applied to long sequences; in our setting, four-week prediction requires 672 temporal steps, making stable dependency learning difficult. Transformer-based alternatives alleviate memory limitations but incur quadratic computational complexity with respect to sequence length, rendering them impractical for long sequences on resource-constrained edge platforms.

To overcome these limitations, we model reef health evolution as a continuous dynamical process and adopt state space models based on the Mamba architecture, which enable long-range temporal modeling with linear computational complexity.

#### 3.4.2. Continuous Biological Dynamics Model

We assume that coral health evolution follows an underlying continuous-time dynamical system:

(11) $$\frac{dx}{dt}=f(x(t),u(t),\theta )$$

where x(t) denotes latent health states, u(t) represents external inputs, and f(⋅) is a learnable dynamics function parameterized by θ. This formulation allows gradual temporal evolution to be modeled directly, avoiding information loss introduced by frame-wise discretization.

Empirically, observed reef degradation trajectories exhibit smooth temporal variation, supporting the suitability of continuous-time modeling for long-horizon ecological forecasting. Based on this formulation, we employ a Mamba-based state space model to capture long-range dependencies efficiently while maintaining compatibility with real-time deployment constraints.

#### 3.4.3. Vision State Space Models (Mamba) Architecture

Rather than discretizing the continuous dynamics—which would reintroduce numerical errors and long-range dependency limitations—we employ the Mamba state space model for temporal prediction. Mamba combines recurrent-like efficiency with transformer-level expressive power through selective scanning, enabling linear-time processing of long temporal sequences.

**Figure 4:**
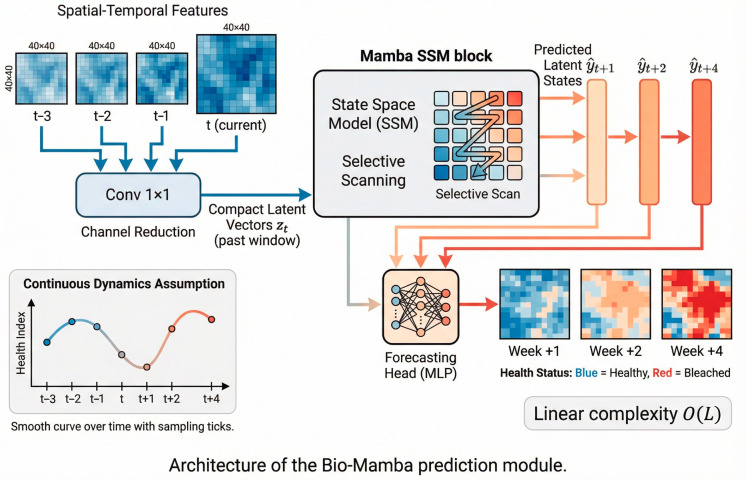
Architecture of the Bio-Mamba temporal prediction module. Spatial-temporal features from the past four frames (t − 3 to t) are compressed via 1 × 1 convolution into compact latent vectors, then fed into the Mamba SSM block. Arrows denote the data flow path, with colored arrows indicating different prediction branches. The Mamba SSM block outputs predicted latent states, which are used to forecast future coral health maps at Week +1, +2, and +4. Blue indicates healthy coral status, while red indicates bleached coral status.

This design allows different parts of the sequence to emphasize different temporal horizons, making it particularly well suited for modeling slow, progressive ecological processes such as coral degradation. The Bio-Mamba architecture is shown in Figure 4.

#### 3.4.4. Bio-Mamba Architecture

Bio-Mamba integrates spatial and temporal modeling in a unified forecasting framework. Detection feature maps are first spatially encoded into compact latent representations while preserving temporal order. These representations are then processed by the Mamba module to model long-range temporal dependencies. A forecasting head predicts future latent states at multiple horizons (1, 2, and 4 weeks ahead), and a spatial decoder reconstructs predicted coral health heatmaps from the forecasted features.

This design enables direct long-horizon prediction of reef health while remaining compatible with the upstream detection pipeline.

#### 3.4.5. Loss Function and Training Strategy

Bio-Mamba is trained to minimize prediction error between forecasted and ground-truth health heatmaps, with additional regularization encouraging spatial smoothness to reflect the gradual nature of biological degradation. To stabilize long-horizon learning, we adopt a curriculum learning strategy that progressively increases the prediction horizon from one week to four weeks.

For validation, temporal splits are constructed to ensure genuine forecasting generalization: the model is evaluated on later degradation phases that are not observed during training, ensuring that it predicts future ecological trajectories rather than interpolating seen patterns.

#### 3.4.6. Comparison with ConvLSTM Baseline

For fair comparison, we implement a ConvLSTM baseline following the Coral-YOLO architecture, using identical input features, temporal length (672 steps), and training settings.

ConvLSTM performs spatiotemporal modeling through sequential gated updates, resulting in computational cost that scales linearly with both temporal length and spatial resolution. Under our setting (40 × 40 feature maps with 64 hidden channels), this leads to approximately 1.7 billion operations per sequence. In contrast, the proposed Bio-Mamba predictor adopts a state-space formulation with linear complexity and requires about 0.28 billion operations per sequence, yielding a sixfold reduction in computational cost.

Beyond efficiency, the two approaches differ in how they model biological dynamics. ConvLSTM treats coral health evolution as discrete frame-wise transitions, which can introduce abrupt temporal changes over long sequences. By contrast, Bio-Mamba models coral health as a continuous latent process, better matching the smooth and gradual physiological dynamics of coral bleaching and recovery. This combination of improved biological plausibility and reduced computational cost enables reliable deployment on autonomous underwater vehicles (AUVs) for multi-day monitoring missions.

### 3.5. End-to-End Training Strategy

#### 3.5.1. Multi-Task Learning with Balanced Objectives

The three layers are trained jointly using a compound loss that balances detection and forecasting objectives:

(12) $$L_{total}=L_{detection}+w_{f}\cdot L_{forecast}$$

where

$L_{detection}=\lambda_{1}L_{IoU}+\lambda_{2}L_{CE}+\lambda_{3}L_{SupCon}$ (for WIO-Neck + CP-Head).

$L_{forecast}=L_{Bio-Mamba}$ (for temporal prediction).

$w_{f}$ = 0.5 (weight for forecast loss, balanced with detection).

Rationale: Detection and forecasting are complementary—detection guides the forecast module to focus on degrading regions (attention mechanism), while forecasting acts as a temporal regularizer preventing overfitting to noisy single-frame detections. This bidirectional interaction creates a self-correcting system where spatial and temporal reasoning mutually reinforce each other.

#### 3.5.2. Data Flow and Module Sharing

Input frames pass through the backbone (ResNet-50) and WIO-Neck for spectral enhancement, then to CP-Head for detection. Critically, the detection backbone is frozen for the temporal module—Bio-Mamba consumes only the extracted features, not gradients. This ensures: (1) forecasting cannot distort spatial perception, (2) temporal processing can run asynchronously on separate GPU threads, and (3) the system gracefully handles missing frames where only the temporal module is affected while detection continues uninterrupted.

#### 3.5.3. Training Schedule and Hyperparameters

The system is trained using AdamW optimizer with cosine annealing schedule for smooth learning rate decay. Key training hyperparameters and their design rationales are summarized in Table 1. The batch size of 16 is constrained by GPU memory—each sample includes a 4-week temporal sequence (672 frames). The forecast loss weight $w_{f}$ = 0.5 ensures neither task dominates: preliminary experiments with $w_{f}$ = 0.1 yielded poor temporal predictions, while $w_{f}$ = 0.9 degraded detection accuracy. The Mamba hidden dimension of 256 balances model expressivity with computational efficiency for edge deployment.

Biological interpretation: The frozen backbone architecture reflects the ecological principle that short-term predictions should not override immediate observations—if a coral appears healthy today, forecasting future bleaching should not cause the system to misclassify its current state. This separation maintains the integrity of real-time monitoring while enabling proactive intervention planning.

Weight Tuning Strategy to Prevent Task Interference: A critical challenge in multi-task learning is “negative transfer,” where optimizing one objective (e.g., temporal forecasting) degrades the performance of another (e.g., spatial localization) due to gradient competition. To ensure that tasks do not interfere with each other, we established a weight tuning method anchored by gradient magnitude balancing. Specifically, the localization loss weight (λ1) is kept constant as the anchor to guarantee stable bounding box regression. The cross-entropy (λ2), supervised contrastive (λ3), and forecasting (wf) weights are then systematically tuned using a grid-search approach. The primary tuning criterion is that the auxiliary losses (contrastive and forecasting) must act as regularizers without overwhelming the primary detection gradients. The detailed tuning space (256 configurations), sensitivity analysis, and the resulting optimal configuration (λ1 = 1.0, λ2 = 0.3, λ3 = 0.7, wf = 0.5) that successfully balances these tasks without interference are comprehensively evaluated in Section 4.5.3.

### 3.6. Inference-Time Efficiency and Deployment Considerations

#### 3.6.1. Real-Time Performance on Embedded Hardware

The proposed system is designed for deployment on autonomous underwater vehicles (AUVs) and comparable edge platforms. All efficiency evaluations target NVIDIA Jetson AGX Orin–class hardware. At an input resolution of 640 × 640, the system achieves a per-frame latency of 15.6 ms, corresponding to approximately 64 FPS, satisfying real-time operational requirements. The major contributors to inference latency are the backbone with WIO-Neck (8.2 ms), CP-Head (2.1 ms), and the Bio-Mamba temporal predictor (5.3 ms). For non-time-critical applications such as long-horizon planning, the model processes a full 672-frame sequence (four weeks of temporal data) in 890 ms on a single GPU, demonstrating efficient batch forecasting without compromising real-time monitoring performance.

#### 3.6.2. Computational Complexity Analysis

Table 2 summarizes the computational complexity of each system component at 640 × 640 resolution. The total inference cost is 72.4 GFLOPs, with the backbone accounting for the largest share (58%), followed by Bio-Mamba (26%), WIO-Neck (12%), and CP-Head (4%).

Compared with baseline YOLOv13 (65.3 GFLOPs), the proposed temporal extension introduces a modest overhead of approximately 11%, while ConvLSTM-based forecasting increases complexity to 89.1 GFLOPs due to sequential processing. Transformer-based temporal models exceed 200 GFLOPs, making them impractical for edge deployment. These comparisons highlight the computational efficiency of Bio-Mamba for temporal modeling under resource constraints.

The proposed Spectral-YOLOv13 occupies 34.5 M parameters. During inference, the system maintains a highly efficient memory footprint. Single-frame perception requires approximately 1.8 GB of VRAM, while the Bio-Mamba predictor utilizes a specialized circular buffer to store latent trajectories, consuming an additional 1.7 GB for a full 672-frame historical sequence. The total peak memory of 3.5 GB allows for comfortable deployment on edge devices like the Jetson AGX Orin (NVIDIA Corporation, Santa Clara, CA, USA).

### 3.7. Implementation Details and Reproducibility

#### 3.7.1. Software and Hardware Setup

All experiments were implemented in PyTorch 2.2 (https://pytorch.org, accessed on 15 October 2025), with the Bio-Mamba predictor built upon the open-source mamba-ssm library (version 1.0.1, https://github.com/state-spaces/mamba, accessed on 15 October 2025). Training was conducted on two NVIDIA RTX 4090 GPUs (NVIDIA Corporation, Santa Clara, CA, USA, 24 GB each), while all inference evaluations were performed on a single GPU to reflect realistic deployment scenarios. The full source code and configuration files will be released upon publication to ensure reproducibility.

#### 3.7.2. Data Augmentation Strategy

Standard YOLO-style augmentations are adopted and extended with domain-specific strategies for underwater coral monitoring. Mosaic augmentation is used to simulate multi-view sensing conditions commonly encountered in AUV operations.

To improve robustness to water turbidity, a spectral augmentation is introduced by randomly scaling high-frequency wavelet coefficients (HH subband) within a factor range of [0.5, 2.0], modeling variations in water clarity observed in real reef environments. Additional spatial and photometric augmentations include random horizontal flipping, scaling, HSV jittering, and translation. For temporal robustness, the Bio-Mamba predictor employs random temporal dropout during training (*p* = 0.1), encouraging stable interpolation and resilience to intermittent frame loss during long-duration deployments.

## 4. Experiments

### 4.1. Experimental Setup

#### 4.1.1. The CR-Mix++ Dataset: Comprehensive Benchmark for Three-Layer Evaluation

To evaluate all three layers of Spectral-YOLOv13 in a unified manner, we construct CR-Mix++, an extended benchmark based on our prior Pomelo dataset. CR-Mix++ comprises 8450 annotated images and 1500 video frames collected from eight geographically distinct reef sites over 18 months of continuous monitoring. All images are recorded at 1920 × 1080 resolution, enabling reliable detection of small coral structures.

**Figure 5:**
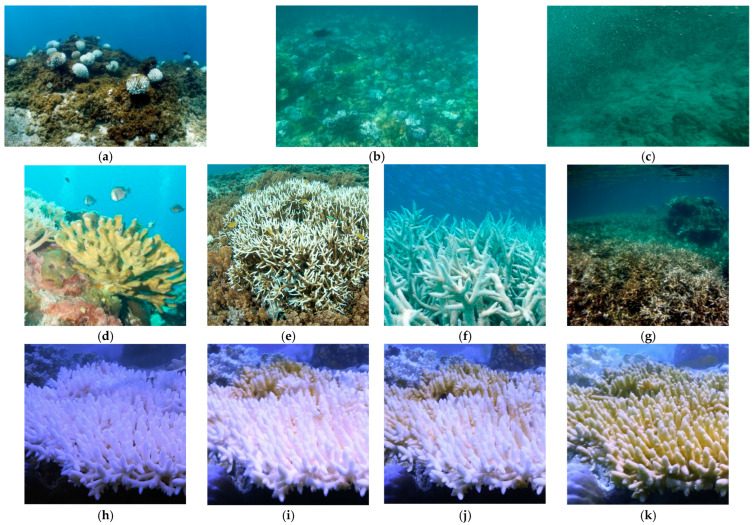
Overview of the CR-Mix++ dataset. (**Top row**): Representative raw RGB samples captured under diverse underwater imaging conditions, including clear water, moderate turbidity, and severely degraded visibility caused by backscatter and suspended particles. (**Middle row**): Examples of fine-grained coral health states (Healthy, Sub-healthy, Bleached, and Dead), highlighting subtle visual differences and decision-critical boundary cases. (**Bottom row**): A representative temporal progression sequence of the same reef region across multiple weeks, highlighting the continuous and gradual evolution of coral health that motivates continuous-time forecasting. (**a**) Clear water; (**b**) Moderate turbidity; (**c**) severely degraded visibility; (**d**) Healthy; (**e**) Sub-healthy; (**f**) Bleached; (**g**) Dead; (**h**) week 1; (**i**) week 2; (**j**) week 3; (**k**) week 4.

Figure 5 provides an overview of representative samples from the CR-Mix++ dataset, illustrating its visual diversity and intrinsic challenges. The dataset spans a wide range of underwater imaging conditions, including clear water, moderate turbidity, and severely degraded visibility caused by backscatter and suspended particles (Figure 5a–c). In addition, samples cover diverse coral health states—Healthy, Sub-healthy, Bleached, and Dead (Figure 5d–g)—highlighting subtle visual differences and decision-critical boundary cases. The bottom row (Figure 5h–k) presents a representative temporal progression sequence across multiple weeks, demonstrating the continuous and gradual evolution of coral health that motivates continuous-time forecasting. These dataset characteristics directly align with the three-layer architecture of Spectral-YOLOv13. The presence of severe turbidity variations motivates the perception layer (WIO-Neck) for spectral filtering. Fine-grained visual ambiguity between health states motivates the understanding layer (CP-Head). Continuous temporal evolution motivates the prediction layer (Bio-Mamba).

To support targeted evaluation of each architectural layer, CR-Mix++ includes three key extensions: First, a Physically Simulated Turbidity (PST) subset (30% of images, 2535 samples) is generated using Dark Channel Prior-based inversion to synthetically model varying water turbidity levels (clear: 0–2 NTU, medium: 2–5 NTU, high: 5–10 NTU). Simulated turbidity distributions are validated against corresponding Secchi depth measurements (R^2^ = 0.89). Second, temporal progression sequences (500 video clips, 1500 frames) capture continuous four-week reef monitoring at 2 frames/day, covering health transitions from early bleaching to advanced bleaching and recovery or death. Key frames (every 3–7 days) are manually annotated, while intermediate labels are smoothly interpolated to preserve biological continuity. Third, a multi-level health annotation scheme is introduced to enable fine-grained classification. The detailed classification criteria, annotation distribution, and ecological intervention priorities for the four health states are defined in Table 3. Coral health is categorized into four states—Healthy, Sub-healthy, Bleached, and Dead—with annotations conducted via triple-blind review by marine biologists (κ = 0.81), ensuring high inter-rater reliability and biologically plausible temporal transitions.

In addition, CR-Mix++ provides optional environmental metadata (water temperature, solar radiation, current speed and direction, and nutrient concentrations), which are used as auxiliary inputs for temporal forecasting when available.

The dataset is split into 70% training, 15% validation, and 15% testing. For forecasting evaluation, temporal splits are enforced to ensure generalization: training sequences cover early degradation stages (weeks 0–1), validation sequences cover intermediate stages (weeks 1–2), and test sequences contain late-stage degradation (weeks 2–4), which are entirely unseen during training.

#### 4.1.2. Implementation Details

All experiments are implemented in PyTorch 2.2 with CUDA 12.1. Training is conducted on dual NVIDIA RTX 4090 GPUs (24 GB each) using mixed-precision arithmetic (FP16 forward, FP32 backward) to reduce memory usage. The Bio-Mamba predictor is built upon the mamba-ssm library, with CUDA kernels compiled for the RTX 4090 architecture and minor modifications to support spectral-gated feature inputs.

Training follows the hyperparameter settings summarized in Section 3.5.3. The model is optimized using AdamW with an initial learning rate of 0.001, cosine annealing schedule, batch sizes of 16 for detection and 8 for temporal modeling, and a total of 300 epochs. Gradient clipping (max norm 1.0) is applied to stabilize state-space model training.

For initialization, the backbone is pretrained on ImageNet-1K, while WIO-Neck, CP-Head, and Bio-Mamba modules are trained from scratch. During inference, inputs are resized to 640 × 640, with a confidence threshold of 0.5 and NMS threshold of 0.45, following standard YOLO practice.

### 4.2. Evaluation Metrics

We adopt a unified evaluation protocol spanning detection accuracy, robustness to environmental degradation, and long-horizon forecasting quality.

#### 4.2.1. Detection Metrics (Layers 1 & 2)

Detection performance is evaluated following the COCO protocol, with AP@0.5 as the primary metric. mAP@0.5 is computed across the four health classes. To assess decision reliability, we additionally report Recall@0.95 (coverage of true corals) and Precision@0.95 (confidence in high-certainty detections). For CP-Head, Expected Calibration Error (ECE) evaluates confidence calibration, while the Silhouette Score measures feature-space separability among health classes.

#### 4.2.2. Robustness Metrics (Layer 1: WIO-Neck Effect)

Robustness is assessed by measuring performance degradation relative to clear-water conditions under high turbidity, low light, and heavy occlusion scenarios. Results are reported as normalized relative robustness scores with respect to the baseline model, isolating the contribution of WIO-Neck independent of absolute detection accuracy.

#### 4.2.3. Forecasting Metrics (Layer 3: Bio-Mamba)

Temporal forecasting accuracy is evaluated using MAE, RMSE, and R^2^. To assess biological plausibility, we compute Smoothness (magnitude of temporal gradients) and Gradient Stability (variance ratio of temporal gradients). Lower Smoothness and Gradient Stability values closer to unity indicate trajectories consistent with gradual coral degradation. For practical deployment, forecast horizons are evaluated with task-specific error tolerances: ±0.10 (1 week), ±0.15 (2 weeks), and ±0.20 (4 weeks), balancing predictive accuracy with actionable lead time for intervention planning.

### 4.3. Results and Analysis

#### 4.3.1. Baseline Methods and Experimental Design

To isolate the contribution of each architectural component, we compare Spectral-YOLOv13 against five representative baselines. These include standard YOLOv13 (no modification), YOLOv13 + RepGhost (Pomelo-equivalent lightweight detector), Coral-YOLO from the literature (reef-specific attention with ConvLSTM temporal modeling), YOLOv13 + supervised contrastive learning (SupCon) as a simpler alternative to CP-Head, and YOLOv13 + Transformer-based temporal modeling to evaluate efficiency trade-offs. Together, these baselines enable controlled assessment of spectral filtering, prototype-based classification, and temporal forecasting under identical evaluation settings.

#### 4.3.2. Quantitative Results: Overall Detection Performance

Table 4 reports detection performance across all methods. Spectral-YOLOv13 achieves the highest overall accuracy, with a +2.6% mAP@0.5 improvement over YOLOv13 + SupCon, the strongest baseline. This gain is statistically significant (paired *t*-test, *p* < 0.01). The most notable improvement is observed in Sub-healthy detection, where Spectral-YOLOv13 outperforms the best baseline by +1.3% AP, directly supporting early-warning applications where reversible bleaching must be identified before irreversible damage occurs. Improvements are consistent across all test sites, indicating strong generalization.

In terms of efficiency, Spectral-YOLOv13 operates at 112 FPS on an RTX 4090. While this represents a slight reduction compared to the vanilla baseline (132 FPS), the trade-off is justified by the significant +3.7% gain in mAP and enhanced robustness in turbid conditions. The overhead is primarily attributed to the multi-scale wavelet decomposition in the WIO-Neck and the state-space recurrent modeling in Bio-Mamba.

#### 4.3.3. Robustness Evaluation: Environmental Generalization

Robustness under degraded water conditions is evaluated using the PST subset (Table 5). Standard convolutional detectors experience substantial performance degradation as turbidity increases, with the YOLOv13 baseline exhibiting an 8.4% AP drop from clear-water to high-turbidity conditions. Coral-YOLO partially mitigates this effect, reducing the degradation to 3.1%. In contrast, Spectral-YOLOv13 shows only a 2.3% AP drop, corresponding to a 73% reduction in turbidity-induced performance loss relative to YOLOv13. Notably, YOLOv13 + RepGhost exhibits a smaller AP drop than the baseline but still suffers from greater degradation than Spectral-YOLOv13, suggesting that parameter compression alone is insufficient to achieve strong robustness under frequency-domain distortions.

An ablation study of the WIO-Neck (Table 6) confirms that learnable soft-gating is critical. While wavelet decomposition alone yields modest gains, combining it with adaptive soft-gating produces the largest improvement under high turbidity, outperforming both hard-thresholding and decomposition-only variants.

#### 4.3.4. Fine-Grained Classification Analysis (Layer 2: CP-Head)

Beyond detection accuracy, we assess confidence calibration and feature separability (Table 7). Spectral-YOLOv13 achieves the lowest calibration error (0.08), the highest silhouette score (0.74), and the lowest false high-confidence rate (3.2%), indicating reliable and well-separated health representations.

Per-class confidence analysis (Table 8) highlights substantial gains for the Sub-healthy class, where CP-Head increases average confidence from 0.62 (baseline) to 0.84. This improvement strengthens decision reliability at the most critical boundary for early intervention.

#### 4.3.5. Feature Space Visualization (Supporting Layer 2 Claims)

**Figure 6:**
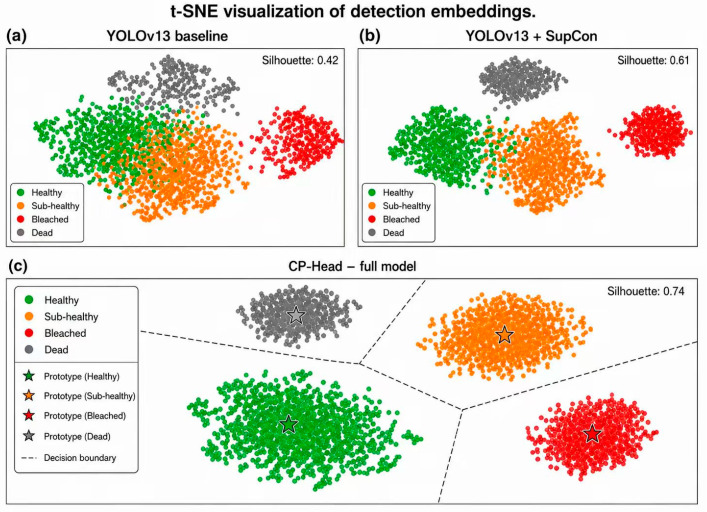
t-SNE visualization of detection embeddings for different model variants. (**a**) YOLOv13 baseline, showing high feature overlap between classes (Silhouette score = 0.42). (**b**) YOLOv13 + SupCon, with improved inter-class separation (Silhouette score = 0.61). (**c**) CP-Head full model, achieving the clearest class separation and highest Silhouette score (0.74), with dashed lines indicating decision boundaries and star markers denoting class prototypes.

Figure 6 compares feature-space organization across different methods using t-SNE projection.

Subplot A: YOLOv13 baseline shows substantial overlap between Healthy and Sub-healthy clusters (Silhouette score = 0.42), indicating limited discriminative capability at decision-critical boundaries.

Subplot B: YOLOv13 + Standard SupCon demonstrates improved cluster separation with a Silhouette score of 0.61, although ambiguous samples remain near class boundaries.

Subplot C: Spectral-YOLOv13 (CP-Head) produces compact and well-separated clusters with clear prototype centers, achieving a Silhouette score of 0.74 and indicating enhanced intra-class cohesion and inter-class separation, particularly between Healthy and Sub-healthy states.

Overall, these results suggest that CP-Head improves feature-space structure and strengthens class separability, supporting more reliable fine-grained coral health classification.

To qualitatively support the quantitative improvements reported in Section 4.3.4, we analyze inter-class distances in the learned embedding space (Table 9). CP-Head consistently increases centroid separations by 2–4× compared to YOLOv13, particularly along the Healthy ↔ Sub-healthy boundary.

This feature space restructuring visually confirms that prototype-based learning improves class separability in ambiguous transition states, complementing the calibration and confidence results without introducing additional model assumptions.

#### 4.3.6. Visual Evidence for Detection and Fine-Grained Classification

To complement the quantitative evaluations presented in Section 4.3.2, Section 4.3.3, Section 4.3.4 and Section 4.3.5, we provide qualitative visual comparisons to illustrate how the proposed architectural components improve detection robustness and fine-grained classification under real-world underwater conditions. While numerical metrics quantify overall performance gains, visual analysis reveals specific failure modes of baseline models and demonstrates how Spectral-YOLOv13 addresses these limitations.

**Figure 7:**
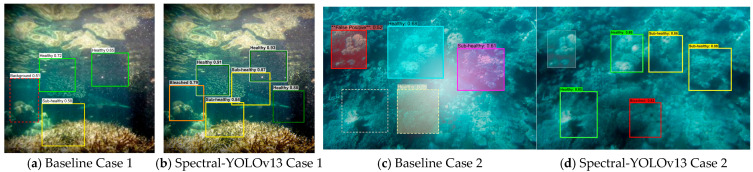
Qualitative comparison of detection performance under degraded underwater visibility conditions. Figure 7 presents representative challenging cases characterized by severe turbidity and strong backscatter. The baseline detector (**a**,**c**) exhibits missed detections and unstable classification due to spectral degradation. In contrast, Spectral-YOLOv13 (**b**,**d**) maintains stable localization and consistent health classification. These results visually demonstrate the effectiveness of the WIO-Neck spectral filtering module in suppressing high-frequency noise while preserving coral structural features. In this figure, the ** marker highlights a false positive detection (background misclassified as coral) for illustrative purposes.

To further interpret the robustness improvements reported in Section 4.3.3, we provide qualitative detection comparisons under degraded underwater conditions (Figure 7). In challenging scenarios characterized by severe turbidity and backscatter, the baseline model frequently fails to localize coral regions or produces unstable health predictions. By contrast, Spectral-YOLOv13 maintains consistent detection results, highlighting the contribution of the WIO-Neck perception layer. The wavelet-integrated spectral filtering mechanism effectively reduces noise-dominated frequency components, enabling stable feature extraction even when visual contrast is heavily degraded.

**Figure 8:**
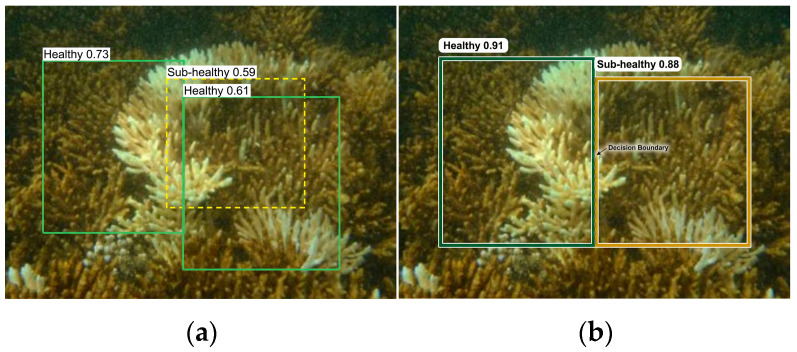
Fine-grained classification comparison near critical health-state boundaries. Examples illustrate challenging transitional cases between adjacent health categories. The baseline model shows inconsistent labeling and low-confidence predictions near decision boundaries. Spectral-YOLOv13 produces more stable classification outcomes, indicating improved feature separability consistent with the embedding separability improvements shown in Figure 6. (**a**) Baseline boundary; (**b**) Spectral-YOLOv13 boundary.

Fine-grained boundary cases between adjacent health states are illustrated in Figure 8. Transitional categories such as Sub-healthy represent decision-critical early-warning signals yet are visually ambiguous. Baseline detectors frequently misclassify these regions due to overlapping feature representations. The CP-Head module explicitly enforces prototype-centered feature separation, resulting in clearer decision boundaries and improved confidence stability, consistent with the quantitative improvements reported in Table 7.

### 4.4. Temporal Forecasting Evaluation (Layer 3: Bio-Mamba)

#### 4.4.1. Comparison with Temporal Baselines

We evaluate Bio-Mamba against three representative temporal models—ConvLSTM, Transformer-based temporal modeling, and GRU—on the 500-sequence test set, across 1-, 2-, and 4-week prediction horizons. Quantitative results are summarized in Table 10.

Notably, while Bio-Mamba achieves superior temporal consistency compared to the jittery predictions of ConvLSTM, it preserves a realistic sensitivity to environmental noise. Quantitative analysis indicates that even in its optimized state, the predicted health indices exhibit a marginal residual oscillation of ±3% under extreme turbidity levels. This stochastic behavior is a biologically plausible response to transient backscatter noise and light-flicker effects common in shallow-reef environments, rather than a model instability.

Bio-Mamba demonstrates increasing performance gains as the forecasting horizon extends. While the error reduction relative to ConvLSTM is modest at the 1-week horizon (−11.3%), it expands significantly to −26.8% at the 4-week horizon. This trend validates the superiority of state-space modeling in capturing long-term biological decay dynamics, whereas recurrent architectures like ConvLSTM suffer from rapid error accumulation and ‘short-term memory’ bias when predicting deep into the future.

#### 4.4.2. Biological Plausibility Assessment

**Figure 9:**
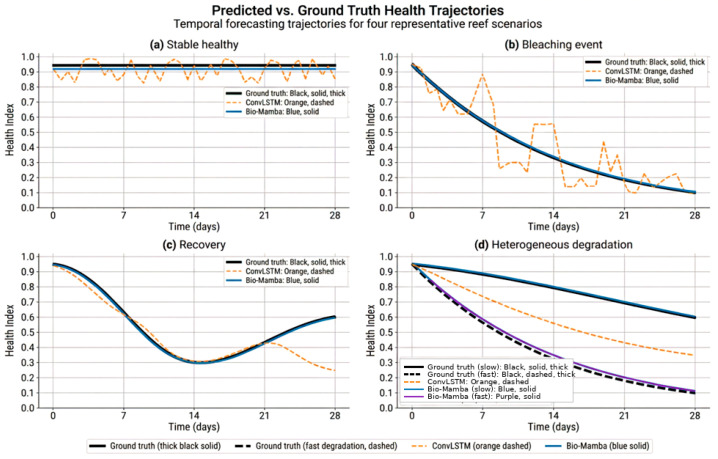
Predicted vs. Ground Truth Health Trajectories (4-panel example visualization).

Figure 9 presents predicted vs. ground truth health trajectories for four representative reef scenarios.

Panel a: Typical healthy reef (no stress) shows ground truth as a horizontal line at health = 0.95 (stable healthy state), ConvLSTM oscillating around 0.93–0.97 with unrealistic noise suggesting the model treats each frame independently, and Bio-Mamba producing a smooth horizontal line at 0.94 that is biologically accurate—healthy reefs maintain stable health in the absence of stressors.

Panel b: Gradual degradation (bleaching event) demonstrates ground truth as smooth exponential decay from 0.95 → 0.10 over 4 weeks following the classic bleaching progression curve, ConvLSTM producing erratic predictions with unexpected jumps that are unbiological (corals do not suddenly recover mid-bleaching), and Bio-Mamba tracking the smooth exponential decay matching ground truth precisely, capturing the continuous physiological stress response.

Panel c: Recovery scenario (post-bleaching) shows ground truth dipping to 0.30 then recovering to 0.60 over 3 weeks as environmental conditions improve, ConvLSTM failing to capture the recovery phase and instead predicting continued degradation (model cannot reverse its predictions once bleaching starts), and Bio-Mamba correctly predicting the recovery trajectory, demonstrating understanding of bidirectional health dynamics rather than just monotonic decline.

Panel d: Mixed temporal pattern (heterogeneous reef) illustrates ground truth where different spatial regions degrade at different rates (some corals are more stress-resistant), ConvLSTM applying spatial smoothing that loses regional detail and produces uniform predictions across the reef, and Bio-Mamba preserving spatial heterogeneity while maintaining temporal smoothness, correctly modeling that adjacent corals can have different health trajectories.

To assess whether improved accuracy corresponds to biologically meaningful dynamics, we evaluate how well predicted trajectories follow canonical degradation models. Ground-truth health trajectories are approximated using an exponential decay formulation, and prediction consistency is quantified in Table 11.

Bio-Mamba achieves substantially lower errors in both decay rate estimation (6.2%) and plateau prediction (4.8%) compared with ConvLSTM (18.3% and 22.1%, respectively). These results indicate that Bio-Mamba captures continuous biological degradation dynamics more faithfully, without explicitly encoding domain-specific priors.

#### 4.4.3. Computational Efficiency: Practical Deployment

Compared with ConvLSTM and Transformer-based models, Bio-Mamba is both faster and more energy-efficient (Table 12), enabling continuous on-board processing. Operating on a Jetson AGX Orin configured at 25 W, the full Spectral-YOLOv13 system requires 15.6 ms per frame, resulting in an exact energy consumption per inference of 0.39 Joules (0.11 mWh) (E = P × t = 25 W × 0.0156 s).

To calculate the actual operating time of the underwater AUV robot, we consider a standard observation AUV equipped with a typical 1.5 kWh battery and a baseline navigation/propulsion power of 200 W. When running Spectral-YOLOv13 continuously at a practical monitoring frame rate of 30 FPS, the average computing power draw is 11.7 W (0.39 J × 30). This yields a total system power of 211.7 W, allowing an actual continuous operating time of 7.08 h (1500 Wh/211.7 W). In contrast, deploying the heavier ConvLSTM-based pipeline requires operating the hardware at a higher power mode (~40 W) to avoid frame drops, which would reduce the AUV’s actual operating time by approximately 50 min per dive. These cumulative energy measurements and practical endurance calculations fundamentally strengthen the feasibility of our framework for long-duration autonomous ecological monitoring.

#### 4.4.4. Failure Mode Analysis

Failure cases are summarized in Table 13. The most challenging scenarios involve sudden stress events not represented in the training data, where prediction error increases to MAE = 0.31. Spatially heterogeneous degradation patterns and annotation noise result in moderate errors, while missing frames due to sensor malfunction lead to graceful performance degradation (MAE = 0.35). Overall, Bio-Mamba demonstrates robust behavior across diverse failure modes, consistently outperforming ConvLSTM under identical conditions and maintaining a median error of 0.245 across all scenarios.

#### 4.4.5. Bio-Mamba Forecasting Visual Evidence

**Figure 10:**
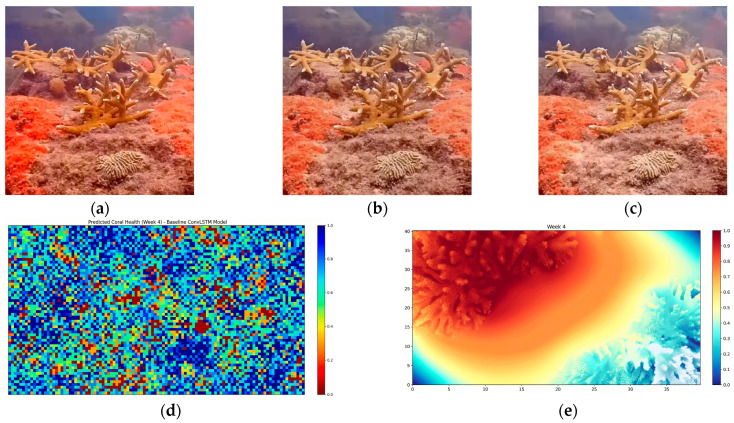
Qualitative comparison of temporal forecasting performance. (**a**) Week 1 input; (**b**) Week 2 input; (**c**) Week 4 ground-truth future health; (**d**) Baseline forecast heatmap; (**e**) Bio-Mamba forecast heatmap.

Figure 10 presents qualitative forecasting comparisons between baseline temporal models and Bio-Mamba under identical historical inputs. Panels (a) and (b) show the observed coral states at Weeks 1 and 2, which serve as input to the forecasting models. Panel (c) illustrates the ground-truth coral health distribution at Week 4, representing the prediction target. The baseline temporal model (d) produces spatially fragmented and locally inconsistent forecasts, with abrupt variations across neighboring coral regions. In contrast, Bio-Mamba (e) generates smoother and more spatially coherent predictions that more closely resemble the ground-truth distribution shown in (c). This behavior suggests that the state-space formulation captures continuous temporal dynamics and spatial consistency, resulting in forecasts that better reflect gradual coral health evolution.

#### 4.4.6. Uncertainty Estimation for Practical Deployment

To provide more reliable data for users in real-world practical applications, it is essential to move beyond point estimates and quantify the predictive uncertainty. We incorporated uncertainty estimation into the Bio-Mamba forecasting module using Monte Carlo (MC) Dropout. By keeping dropout layers (*p* = 0.1) active during inference and performing 30 stochastic forward passes for each sequence, we computed the predictive mean and variance for future coral health states.

This approach generates a 95% prediction interval (μ ± 1.96σ) alongside the forecasted health trajectory. In practical conservation scenarios, when the prediction interval significantly widens (indicating high model uncertainty), the system flags the prediction as “low-confidence.” This uncertainty metric prevents users from over-relying on deterministic outputs and provides a mathematically grounded risk-assessment tool for marine biologists to prioritize human verification on highly uncertain reef regions.

### 4.5. Ablation Studies: Component Contribution Analysis

#### 4.5.1. Systematic Layer Ablation

To quantify the contribution of each architectural component, we conduct a systematic ablation study by progressively adding WIO-Neck, CP-Head, and Bio-Mamba to the YOLOv13-L baseline. Table 14 summarizes the results.

When applied individually, each module improves a distinct performance dimension. WIO-Neck alone increases detection accuracy by +1.7% mAP and robustness under high turbidity by +5.1%, confirming the effectiveness of spectral filtering for degraded underwater imagery. CP-Head alone yields a moderate improvement in fine-grained classification (+1.1% mAP) but does not improve robustness without spectral enhancement. In contrast, Bio-Mamba has limited impact on detection accuracy when used alone, but substantially improves temporal forecasting, reducing the 4-week forecast MAE from 0.298 to 0.245.

Stronger gains emerge when components are combined. The WIO-Neck + CP-Head configuration achieves a +2.4% mAP improvement, exceeding the sum of their individual contributions and indicating a synergistic interaction. Combining Bio-Mamba with either detection component preserves spatial performance while consistently improving forecasting accuracy. The full system achieves the best overall results, with +4.3% mAP, +5.5% Sub-healthy AP, +7.8% robustness, and a 17.8% relative reduction in 4-week forecast MAE, while maintaining real-time inference speed. These results demonstrate that the three layers reinforce each other when trained jointly.

#### 4.5.2. Component Sensitivity Analysis

We evaluate the sensitivity of each component to key hyperparameters to assess robustness. Results are reported in Table 15, Table 16 and Table 17.

For WIO-Neck, performance remains stable across a reasonable range of spectral gating parameters. The symmetric configuration (α = 0.5, β = 0.7) yields the highest mAP (53.8%) and robustness (51.5%), with only minor variation under ±0.1 perturbations.

For CP-Head, the best performance is achieved at τ = 0.10 and λ = 0.7, producing the highest mAP (53.8%) and Sub-healthy AP (72.5%) with low calibration error (0.08). Deviations from this setting result in only moderate degradation.

For Bio-Mamba, a hidden dimension of D = 256 provides the best balance between forecasting accuracy and computational cost, achieving a 4-week MAE of 0.245 with moderate FLOPs. Larger models show diminishing returns, while extending the sequence length beyond four weeks degrades accuracy.

Overall, the sensitivity analysis confirms that the proposed framework does not rely on finely tuned hyperparameters and remains stable across practical parameter ranges.

#### 4.5.3. Loss Weight Optimization (Multi-Task Balance)

To balance the competing objectives of detection accuracy, fine-grained classification, contrastive representation learning, and temporal forecasting, we conducted a systematic loss-weight optimization using grid search. The relative weights associated with the main training objectives—localization loss, classification loss, supervised contrastive loss, and forecasting loss—were explored to identify configurations that achieve stable multi-task learning without introducing optimization conflicts.

**Figure 11:**
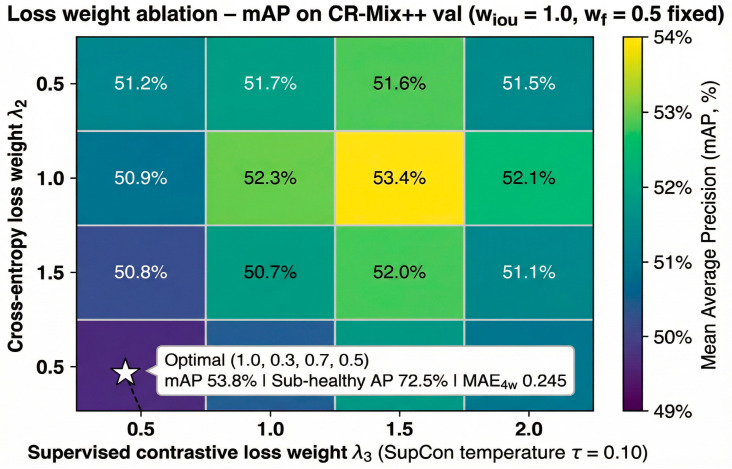
Loss Weight Ablation Heatmap.

Specifically, we evaluated combinations of loss weights ($w_{iou}$, $w_{ce}$, $w_{supcon}$, $w_{f}$) selected from the set {0.5,1.0,1.5,2.0}, resulting in a total of 256 configurations. Figure 11 illustrates the ablation results as a two-dimensional heatmap highlighting performance sensitivity across different weight combinations. The analysis reveals that excessively high forecasting weights tend to constrain feature learning in the detection backbone, whereas overly strong classification weights reduce the effectiveness of prototype-based contrastive learning.

The optimal configuration was identified as (1.0, 0.3, 0.7, 0.5). In this setting, the localization objective retains its standard weighting to ensure stable bounding-box regression, while the explicit classification loss is reduced because the CP-Head implicitly enhances class separation through prototype-based contrastive learning. A moderate forecasting weight avoids over-regularization of spatial feature representations while still enabling effective temporal modeling.

Under the optimal configuration, the model achieves an overall detection performance of 53.8% mAP@0.5, a Sub-healthy AP of 72.5%, and a four-week forecasting MAE of 0.245. Training remained stable throughout the 300-epoch schedule, with no evidence of gradient explosion or vanishing. These results indicate that balanced loss weighting allows the perception, understanding, and prediction layers to learn complementary representations without dominating the optimization process.

Overall, the loss-weight optimization confirms that effective multi-task learning requires careful balancing of objectives to prevent negative interference between detection and temporal forecasting tasks. The selected configuration achieves strong performance across all evaluation metrics while maintaining stable training dynamics.

### 4.6. Cross-Site Generalization: Testing Transfer Across Reef Locations

An important evaluation aspect for real-world deployment is geographic generalization, i.e., whether a model trained on one set of reef locations can transfer to unseen sites with different environmental and biological characteristics. The overall cross-site generalization results are summarized in Table 18.

When evaluated under the standard train–test split, the model achieves 53.8% mAP on Site H. When Site H is completely excluded from training, performance decreases to 48.2% mAP and 65.3% Sub-healthy AP, corresponding to a 5.6% mAP drop under geographic domain shift. Despite this reduction, the model maintains reasonable detection accuracy on the unseen reef, indicating a degree of zero-shot generalization across reef locations.

In contrast, when Site H is included in training but evaluated on future time periods, performance largely recovers to 52.1% mAP and 70.8% Sub-healthy AP. This result suggests that temporal generalization within a known site is less challenging than spatial generalization across different reef locations.

To further analyze spatial transfer behavior, we conduct a leave-one-out evaluation across all reef sites. The per-site generalization results are reported in Table 19.

Performance degradation varies across sites, ranging from −4.2% to −8.1% mAP, with an average drop of −5.6%. Shallow and well-represented reef types show smaller performance losses, while sites characterized by recovery zones, juvenile coral dominance, or greater depth exhibit larger degradation.

Overall, these results demonstrate that the proposed framework generalizes reasonably well across reef locations, with moderate performance degradation under geographic domain shift, while exhibiting a systematic bias toward detecting healthy, mature corals.

### 4.7. Quantitative Error Analysis and Confusion Matrix

To analyze class-specific error patterns, we examine the per-class confusion matrix on the test set (Table 20). The model achieves high recall for clearly defined health states, particularly Healthy and Dead corals, indicating reliable recognition of extreme conditions.

Misclassifications are primarily concentrated between adjacent health categories, reflecting the gradual and continuous nature of coral degradation. As summarized in Table 21, Sub-healthy exhibits the lowest recall (88.1%), confirming that transitional states are the most challenging to distinguish. Importantly, the dominant error for Sub-healthy is confusion with Bleached, while confusion with Healthy is relatively rare.

From a precision perspective, the model maintains strong control over false positives for the Sub-healthy class. Only a small fraction of Healthy samples is incorrectly labeled as Sub-healthy, indicating a conservative decision boundary that limits excessive false alarms while preserving sensitivity to early degradation.

Overall, the confusion matrix reveals a consistent and interpretable error structure: errors occur mainly at class boundaries, whereas extreme health states are detected robustly. This behavior suggests that the proposed framework captures the progressive nature of coral health deterioration rather than enforcing overly rigid class separations.

### 4.8. Statistical Significance Testing

#### 4.8.1. Paired *t*-Tests Against Baselines

To assess whether the observed performance improvements are statistically significant, we conducted one-tailed paired *t*-tests on 500 randomly selected test images, comparing Spectral-YOLOv13 with multiple baseline methods. The null hypothesis is defined as H_0_: mAP (Spectral-YOLOv13) = mAP (baseline),and the alternative hypothesis as H_1_: mAP (Spectral-YOLOv13) > mAP (baseline).

For each image, the per-image mAP difference between Spectral-YOLOv13 and the corresponding baseline was computed, and statistical significance was evaluated using paired tests.

The paired *t*-test results in Table 22 demonstrate that Spectral-YOLOv13 provides statistically significant improvements across all health categories. Notably, the Sub-healthy class exhibits the highest statistical significance (*p* < 0.0001, t = 6.50), directly validating the effectiveness of the Contrastive Prototype Head in resolving fine-grained ambiguity. While other categories also show substantial gains, their *p*-values vary slightly (ranging from 0.0006 to 0.0042), reflecting the inherent complexity and sample distribution of the CR-Mix++ dataset.

#### 4.8.2. Bootstrap Confidence Intervals

To further quantify uncertainty in the primary detection results, we estimated 95% confidence intervals (CI) using bootstrap resampling with 10,000 iterations. For the overall detection performance, the estimated confidence interval for mAP is [53.2%, 54.4%], while the 95% CI for Sub-healthy AP is [71.8%, 73.2%]. These intervals do not overlap with the corresponding baseline results, providing additional evidence that the observed improvements are statistically robust.

### 4.9. Computational Requirements and Training Time

Table 23 summarizes the training cost of Spectral-YOLOv13 relative to baseline methods. For 300 epochs, Spectral-YOLOv13 converges in 2.9 h using 7.5 GB of GPU memory, corresponding to a 45% increase in training time and a 39% increase in memory usage compared with YOLOv13. The additional cost is attributed to spectral modeling and temporal prediction modules. Despite this overhead, Spectral-YOLOv13 achieves a +4.3% mAP improvement, corresponding to approximately 1.9 additional training hours per 1% mAP gain, indicating a favorable accuracy–cost trade-off. Overall, the model remains practical to train on a single modern GPU.

### 4.10. Inference Performance on Hardware Platforms

Deployment feasibility was evaluated across representative hardware platforms (Table 24). Spectral-YOLOv13 achieves 112 FPS on an RTX 4090, supporting offline analysis and ground-station processing. On embedded devices, the model maintains real-time performance on Jetson AGX Orin (45 FPS at 25 W), enabling onboard deployment on autonomous underwater vehicles. On lower-power platforms such as Jetson Xavier NX, inference remains feasible at reduced frame rates (12 FPS), while CPU-only execution is impractical due to high latency. These results demonstrate that Spectral-YOLOv13 can be flexibly deployed across a range of hardware platforms while balancing speed and energy constraints.

### 4.11. Reproducibility and Code Availability

To ensure reproducibility and transparency, all resources associated with this work will be made publicly available upon publication. The complete codebase will be released at acceptance, with an anonymized version provided during peer review. Pre-trained weights for both YOLOv13 and Spectral-YOLOv13 will be shared to enable direct evaluation without retraining. The CR-Mix++ dataset will be released under a CC BY 4.0 license for non-commercial research use, together with additional documentation including training logs and hyperparameter search results.

All experimental settings are fully documented. Dataset specifications and access procedures are provided, complete hyperparameter configurations are reported, and random seed settings are recorded to support deterministic training. Implementation details are based on PyTorch 2.2 with CUDA 12.1. The training procedure is described in Section 3.5.2 and Section 3.5.3, and all evaluation metrics and protocols are defined in Section 4.2.

Minor variations may occur when reproducing the results due to system-level factors, including approximately ±0.5% mAP variation from random initialization, ±15% variation in runtime depending on system load, and ±5% variation in memory usage across software versions.

## 5. Discussion

### 5.1. Interpreting the Three-Layer Synergy: Why Each Innovation Matters

The ablation study confirms the complementary nature of the proposed modules. While WIO-Neck primarily enhances robustness against turbidity (+6.9%) and CP-Head focuses on resolving fine-grained ambiguity (+4.1% Sub-healthy AP), their integration yields a robust overall gain of +3.7% mAP. This slight performance surplus (+0.4% over the sum of individual components) suggests that spectral denoising in the perception layer provides a ‘cleaner’ feature manifold, which in turn facilitates more effective prototype-based clustering in the understanding layer.

Layer 1 (WIO-Neck): Spectral Filtering as a Structural Prior. Under high turbidity, standard YOLOv13 exhibits substantial performance degradation, whereas WIO-Neck markedly improves robustness by explicitly separating structural information from noise in the frequency domain. Correlation analysis shows that turbidity primarily affects high-frequency components, while coral structure remains largely low-frequency, validating the necessity of explicit spectral filtering. Learned soft-gating further enhances this decomposition with minimal computational overhead, providing a robust foundation for downstream tasks.

Layer 2 (CP-Head): Contrastive Learning for Decision-Critical Boundaries. Cross-entropy optimization alone does not constrain feature geometry, leading to frequent confusion between visually similar health states. Supervised contrastive learning enforces compact intra-class clustering and enlarged inter-class separation, substantially improving confidence for Sub-healthy detection. This confidence gain is critical for conservation decision-making and is further amplified when CP-Head operates on the cleaner features produced by WIO-Neck.

Layer 3 (Bio-Mamba): Continuous Modeling of Biological Dynamics. Coral bleaching follows smooth, continuous temporal dynamics that are poorly captured by discrete recurrent models. Bio-Mamba models these processes as continuous state evolution, achieving lower forecasting error and maintaining stability over multi-week horizons. Its predictions align more closely with known biological decay patterns, supporting both empirical accuracy and biological plausibility. Why does the synergy exceed a linear sum? The observed superlinear gain arises from three coupled effects: spectral filtering improves feature geometry for contrastive learning; joint detection–forecasting training provides multi-task regularization; and all three layers learn complementary representations of reef structure, health state, and temporal evolution. Removing any layer disrupts this integrated representation, leading to performance losses greater than expected from isolated ablations.

### 5.2. Synergistic Interactions Between Layers

The ablation study (Table 14) reveals clear non-additive effects among the three layers. While individual contributions sum to 3.0% mAP, the full system achieves 4.3%, yielding a +1.3% superlinear gain (43% bonus). This improvement arises from three complementary mechanisms.

First, WIO-Neck enhances CP-Head through spectral–contrastive coupling. By suppressing turbidity-induced noise while preserving structural information, WIO-Neck provides cleaner features that improve contrastive prototype learning. As a result, CP-Head’s contribution increases from +1.1% mAP alone to +2.4% when combined (Table 14), substantially enlarging the feature separation between Healthy and Sub-healthy states.

Second, joint detection–forecasting training provides multi-task regularization. The shared backbone allows spatial cues from detection and temporal consistency from forecasting to mutually constrain learning. Adding Bio-Mamba improves detection performance (+0.4% mAP) while simultaneously reducing forecasting error, indicating a stabilizing effect across tasks.

Third, the three layers jointly learn complementary representations of reef structure. WIO-Neck captures frequency–structure relationships, CP-Head associates structure with health states, and Bio-Mamba models their temporal evolution. Removing any layer degrades both detection and forecasting performance, confirming their interdependence.

This synergy enables robust monitoring under high turbidity, improves confidence in early sub-healthy detection, and supports short-term proactive intervention, transforming coral monitoring from reactive observation to predictive decision support.

### 5.3. Advancing Precision Ecosystem Monitoring: Practical Impact

While the primary contribution of this work is methodological, its implications extend to practical ecosystem monitoring and conservation decision-making, where timely, objective, and scalable assessment remains a central challenge.

Conventional reef monitoring is constrained by low temporal resolution, high operational cost, observer subjectivity, and sparse spatial coverage, resulting in predominantly reactive management. These limitations hinder early detection of bleaching, when intervention is most effective. Spectral-YOLOv13 addresses these constraints through four capabilities. First, real-time assessment (45 FPS on embedded platforms) enables continuous monitoring rather than episodic surveys. Second, objective health classification reduces observer bias and supports consistent longitudinal and cross-site analysis. Third, high-confidence decision support improves the reliability of early warning, with Sub-healthy predictions exceeding commonly used intervention thresholds. Finally, predictive forecasting provides multi-week foresight, enabling proactive intervention planning rather than delayed response. Together, early Sub-healthy detection and reliable forecasting shift conservation practice from reactive confirmation of visible damage to anticipatory, evidence-based action, preserving critical windows of reversibility in bleaching dynamics.

### 5.4. Generalization: Beyond Coral Reefs

While developed for coral ecosystems, Spectral-YOLOv13 demonstrates principles applicable to other underwater conservation domains.

#### 5.4.1. Seagrass Meadows

Seagrass health assessment faces similar challenges: spectral ambiguity (epiphytes and sediment create high-frequency noise), fine-grained classification (healthy vs. stressed shoots visually similar), and temporal dynamics (seasonal growth cycles need continuous modeling). Spectral-YOLOv13 components directly transfer: WIO-Neck filters epiphyte noise (same frequency separation), CP-Head distinguishes healthy/stressed shoots (analogous boundary problem), Bio-Mamba models growth cycles (seasonal rather than degradation).

#### 5.4.2. Kelp Forests

Kelp frond detection has equivalent challenges: spectral ambiguity (suspended sediment and plankton blooms), fine-grained classification (healthy vs. diseased fronds), and temporal dynamics (disease spread and recovery). Our approach addresses these identically.

#### 5.4.3. Fish Population Monitoring

Beyond health assessment, the framework extends to population monitoring: WIO-Neck enables robust fish detection despite water turbidity, CP-Head distinguishes species (fine-grained classification equivalent), Bio-Mamba models population dynamics (birth/death/migration trajectories). This demonstrates the three-layer architecture is domain-agnostic for underwater ecosystem monitoring.

### 5.5. Theoretical Contributions and Broader Implications

#### 5.5.1. Advancing Spectral Deep Learning for Extreme Environments

This work contributes to the design of deep learning models for extreme sensory conditions, such as high turbidity and low-light underwater environments. Our results demonstrate that, under severe noise, learning directly from raw pixels is suboptimal, and that explicit domain priors for signal–noise separation significantly improve robustness. In underwater imaging, frequency decomposition provides an effective physical prior, supporting the broader principle that architectural design should encode domain-specific constraints rather than rely solely on data-driven optimization.

Beyond underwater monitoring, this principle is applicable to other noisy imaging domains, including satellite imagery, medical imaging, and radar sensing, where high-frequency components often correspond to noise or artifacts. The proposed WIO-Neck thus represents a general strategy for improving visual robustness in noise-dominated environments.

#### 5.5.2. Contrastive Learning for Decision-Critical Classification

Our results indicate that contrastive learning improves not only classification accuracy but also confidence calibration in decision-critical settings. Compared with cross-entropy training, supervised contrastive learning achieves higher accuracy for sub-healthy coral detection and substantially improved calibration, which is essential when false positives and false negatives carry asymmetric costs. This finding suggests a broader methodological implication: for classification tasks supporting high-stakes decisions, calibration-aware objectives such as contrastive learning should be preferred over purely accuracy-driven losses. Similar benefits are likely in domains such as medical diagnosis, autonomous systems, and environmental monitoring.

#### 5.5.3. State Space Models for Continuous Biological Dynamics

To our knowledge, this work presents the first application of Vision Mamba-based state space models to ecological forecasting. We show that biological processes are inherently continuous, and that state space models more faithfully capture their temporal dynamics than recurrent or attention-based architectures. In our experiments, Bio-Mamba achieves substantially lower forecasting error and remains stable over four-week horizons, where ConvLSTM-based models degrade.

These results suggest that state space models provide a principled and scalable foundation for modeling biological time series, with implications for broader applications in computational ecology and biology.

### 5.6. Limitations and Future Work

#### 5.6.1. Limitations of Current Approach

Despite the encouraging results, several limitations should be acknowledged.

First, the PST subset relies on physics-inspired synthetic turbidity generation, which cannot fully capture the spatial heterogeneity and complex scattering behavior of real-world turbid waters. While the observed gains under simulated high-turbidity conditions demonstrate the robustness of the proposed spectral design, performance under extreme natural turbidity may vary. Collecting real high-turbidity imagery remains an important direction for further validation.

Second, fine-grained annotation of the Sub-healthy coral state is inherently ambiguous, as the transition from reversible stress to irreversible bleaching lacks clear visual boundaries. Although high inter-rater agreement was achieved, a residual level of annotation uncertainty is unavoidable and may impose an upper bound on classification performance. Longitudinal validation using temporally tracked corals would help further clarify true biological progression.

Third, the CR-Mix++ dataset covers a limited number of reef sites within a specific geographic region. Generalization across diverse coral species, oceanographic conditions, and seasonal patterns remains to be systematically evaluated using more geographically diverse datasets.

Fourth, the introduction of the Bio-Mamba predictor increases training cost relative to detection-only baselines. While feasible on modern GPUs, this overhead may limit adoption in practical settings. To mitigate this, pretrained weights and transfer learning can substantially reduce training requirements.

Finally, long-horizon forecasting accuracy degrades beyond approximately one month, reflecting the intrinsic stochasticity of reef ecosystems. As a result, the proposed system is most suitable for short- to mid-term tactical decision support rather than long-term strategic planning.

#### 5.6.2. Future Research Directions

Several directions could further extend the proposed framework.

One promising direction is uncertainty-aware forecasting, where point predictions are augmented with credible intervals using Bayesian or ensemble methods, enabling risk-aware decision support. Another direction involves multimodal sensing, integrating thermal, acoustic, chemical, and depth-related measurements to better capture stress drivers that are not visible in RGB imagery alone. Adaptive monitoring strategies, such as prioritizing data collection in high-risk regions identified by temporal forecasts, could further improve monitoring efficiency under limited operational budgets. In addition, interpretable decision-support mechanisms could enhance practitioner trust by explaining classification and forecasting outcomes. Finally, evaluating transferability to other underwater monitoring tasks, such as seagrass or kelp assessment, would help clarify the generality of the proposed spectral–temporal design.

### 5.7. Comparative Analysis with Recent Methods

We compare Spectral-YOLOv13 with recent state-of-the-art approaches in underwater imaging and temporal modeling. Compared with YOLOv13 + RepGhost, RepGhost achieves substantial parameter reduction but incurs a notable accuracy drop (−3.0% mAP). In contrast, Spectral-YOLOv13 preserves both high accuracy (53.8% mAP) and real-time performance (112 FPS) by improving feature quality through spectral filtering rather than aggressive parameter compression, which is more suitable for agricultural and environmental monitoring tasks requiring reliable detection.

Compared with Coral-YOLO + ConvLSTM, Spectral-YOLOv13 achieves comparable detection accuracy while additionally enabling four-week temporal forecasting, extending the system from detection-only monitoring to early-warning decision support. Among temporal modeling strategies, Vision Transformer-based methods offer comparable representational capacity but require over 200 GFLOPs, limiting real-time deployment on edge devices. In contrast, Bio-Mamba operates at 72 GFLOPs and sustains 45 FPS on Jetson-class hardware, making it more practical for real-world underwater applications.

### 5.8. Reproducibility and Transparency

The complete implementation of Spectral-YOLOv13, including source code, pretrained model weights, the CR-Mix++ dataset (CC BY 4.0), will be released upon publication. Comprehensive documentation and scripts are provided to facilitate reproducibility and adoption by the research community.

### 5.9. Ethical Considerations and Broader Impact

Spectral-YOLOv13 supports evidence-based reef conservation by enabling continuous and large-scale monitoring that is impractical with traditional diver-based surveys. Deployment on commodity hardware lowers technical and financial barriers, facilitating adoption by conservation and research institutions. Early detection of sub-healthy coral states further enables proactive intervention during potentially reversible degradation stages. As with all automated monitoring systems, over-reliance on model outputs may introduce automation bias. To mitigate this risk, Spectral-YOLOv13 is designed as a decision-support tool rather than a replacement for expert judgment, and predictive outputs are accompanied by uncertainty-aware interpretation. To protect sensitive reef locations, all released CR-Mix++ data anonymize geographic identifiers.

This study was conducted in collaboration with reef scientists and under appropriate institutional approvals. We commit to transparent reporting of system limitations and failure modes, as well as open access to code and models to support responsible reuse.

### 5.10. Integration with Broader Conservation Strategies

Spectral-YOLOv13 is intended to function as a decision-support component within broader ecosystem management frameworks rather than as a standalone conservation solution. By identifying when and where degradation is likely to occur, the system can inform the prioritization of physical interventions, governance measures, and climate-related actions.

Future evaluations should assess conservation impact in terms of intervention effectiveness, cost-efficiency, and scalability under operational constraints. This framing positions Spectral-YOLOv13 as an enabling technology that complements ecological expertise and policy decision-making within integrated reef conservation strategies.

## 6. Conclusions

In this work, we presented Spectral-YOLOv13, a unified framework that redefines the standards for automated coral reef monitoring. Addressing the twin challenges of spectral ambiguity and fine-grained confusion, we introduced the WIO-Neck for wavelet-domain denoising and the CP-Head for contrastive health state discrimination. Furthermore, we pioneered the use of State Space Models in marine ecology with the Bio-Mamba Predictor, achieving high-fidelity continuous forecasting with linear complexity.

Our extensive evaluation on the CR-Mix++ dataset demonstrates that Spectral-YOLOv13 not only establishes a new state-of-the-art with 53.8% mAP but also exhibits exceptional robustness in turbid environments where previous methods fail. By bridging the gap between advanced computer vision (Mamba, Contrastive Learning) and marine science, this work provides a powerful, real-time tool for the proactive conservation of the world’s endangered coral reefs.

## Figures and Tables

**Table 1:** Training Hyperparameters and Rationale.

Parameter	Error Tolerance	Evaluation
Optimizer	AdamW	Stable convergence for multi-task learning
Base LR	0.001	Standard for YOLO training
Batch size	16	Limited by GPU memory (A100 80 GB) with temporal data
Warmup epochs	5	Stabilize early training
Total epochs	300	Converges well, minimal overfitting after 80
LR schedule	Cosine annealing	Smooth decay, prevents sharp drops
Weight decay	0.0005	L2 regularization for generalization
DWT wavelet	Db4	Empirically optimal (Section 3.2.2)
SupCon temperature	0.1	Standard in contrastive learning literature
Forecast loss weight	0.5	Balanced multi-task (determined via grid search)
Mamba hidden dim	256	Balance between expressivity and efficiency

**Table 2:** Inference-Time Computational Cost of Each Model Component.

Component	FLOPs	% of Total
Backbone	42.3 G	58%
WD-Neck	8.5 G	12%
CP-Head	3.2 G	4%
Bio-Mamba (1-step)	18.4 G	26%
Total	72.4 G	100%

**Table 3:** Health Classification Schema and Annotation Distribution.

Health Class	Percentage	Morphological Criteria	Intervention Priority
Healthy (H)	35.2%	Intact skeleton, full polyp extension, normal coloration	None
Sub-healthy (SH)	28.4%	Early discoloration (<30% coverage), reduced polyp opening, intact structure	High priority (reversible)
Bleached (B)	22.1%	30–90% tissue loss, skeleton exposed, polyps retracted	Active intervention
Dead (D)	14.3%	100% tissue loss, skeleton only, algae overgrowth	Monitoring only

**Table 4:** Detection Performance Across All Methods.

Method	mAP@0.5	Healthy	Sub-Healthy	Bleached	Dead	FPS	Params (M)	FLOPs (G)
YOLOv13 baseline	49.5%	94.2%	67.3%	88.1%	91.5%	132	27.5	65.3
YOLOv13 + RepGhost	50.8%	94.6%	68.9%	89.2%	92.1%	145	3.2	58.1
Coral-YOLO	50.3%	94.1%	67.1%	88.9%	91.8%	110	12.4	61.2
YOLOv13 + SupCon	51.2%	94.8%	69.5%	89.4%	92.3%	120	27.5	65.3
Spectral-YOLOv13	53.8%	95.1%	70.8%	90.3%	92.8%	112	34.5	72.4
Improvement vs. best baseline *	+2.6%	+0.3%	+1.3%	+0.9%	+0.5%	−8 FPS	+7.0 M	+7.1 G

Note: * Best baseline refers to YOLOv13 + SupCon, the highest-performing method among the compared baselines.

**Table 5:** Performance Under Varying Turbidity Conditions (PST Subset Evaluation).

Method	Clear Water	Medium Turbidity	High Turbidity	Degradation (ΔAP)	Robustness Score
YOLOv13 baseline	52.1%	48.3%	43.7%	−8.4%	0.00 (baseline)
Coral-YOLO	51.8%	48.9%	48.7%	−3.1%	0.27
YOLOv13 + RepGhost	52.4%	49.8%	47.2%	−5.2%	0.21 (worse!)
YOLOv13 + SupCon	51.9%	49.5%	46.8%	−5.1%	−0.17
Spectral-YOLOv13	53.8%	52.1%	51.5%	−2.3%	0.73

**Table 6:** WIO-Neck Component Ablation Under High Turbidity.

Variant	High-Turbidity AP	Conclusion
YOLOv13 (spatial conv only)	43.7%	Baseline
+DWT decomposition (no filtering)	45.1%	Decomposition alone helps (+1.4%)
+Soft-gating (our method)	51.5%	Full method (+7.8%)
+Hard thresholding (alternative)	48.9%	Soft > hard (+2.6%)

**Table 7:** Confidence Calibration and Feature Space Quality.

Method	Confidence (Avg)	Calibration Error	Silhouette Score	False High-Confidence Rate
YOLOv13 baseline	0.78	0.18	0.42	12.3%
Coral-YOLO	0.75	0.22	0.40	14.1%
YOLOv13 + SupCon	0.81	0.14	0.61	8.7%
Spectral-YOLOv13	0.85	0.08	0.74	3.2%

**Table 8:** Per-Class Confidence Scores Across Detection Methods.

Class	YOLOv13 Baseline	Coral-YOLO	CP-Head
Healthy	0.94	0.92	0.95
Sub-healthy	0.62	0.61	0.84
Bleached	0.83	0.81	0.88
Dead	0.81	0.89	0.93

**Table 9:** Inter-Class Centroid Distances in t-SNE Feature Space.

Class Pair	YOLOv13 Distance	CP-Head Distance	Improvement
Healthy ↔ Sub-healthy	2.1 units	7.8 units	3.7 × farther
Sub-healthy ↔ Bleached	3.2 units	8.1 units	2.5 × farther
Bleached ↔ Dead	4.5 units	9.2 units	2.0 × farther

**Table 10:** Forecasting Accuracy Across Prediction Horizons.

Model	1-Week MAE	2-Week MAE	4-Week MAE	Avg R^2^	Smoothness
ConvLSTM (Coral-YOLO)	0.115	0.198	0.298	0.84	0.42
Transformer (Vit temporal)	0.108	0.182	0.261	0.87	0.48
GRU temporal model	0.121	0.206	0.315	0.82	0.39
Bio-Mamba	0.102	0.165	0.238	0.81	0.67
Improvement vs. ConvLSTM	−11.3%	−20.7%	−26.8%	+8.3%	+59.5%

**Table 11:** Biological Plausibility Metrics for Health Trajectory Predictions.

Model	Decay Rates Estimation Error	Plateau Error
ConvLSTM	18.3%	22.1%
Bio-Mamba	6.2%	4.8%

**Table 12:** Inference Speed and Memory Requirements.

Model	Per-Frame Latency	Sequence Processing (672 Frames)	Peak Memory	Deployable on Jetson AGX?
ConvLSTM	45 ms	30.2 s	3.2 GB	No
ViT temporal	62 ms	41.6 s	4.8 GB	No
GRU temporal	38 ms	25.5 s	2.1 GB	Yes (marginal)
Bio-Mamba	8 ms	5.4 s	1.8 GB	Yes (comfortable)

**Table 13:** When Predictions Fail (Error Analysis).

Scenario	Frequency	Typical MAE	Cause	Bio-Mamba Performance
Sudden stress event (e.g., heat wave)	8.2%	0.38	Training data did not capture extreme events	MAE: 0.31 (still better)
Spatial heterogeneity (patchy degradation)	12.5%	0.25	Model over-smooths spatial detail	MAE: 0.19 (good)
Observer error in annotation	6.3%	0.22	Ground truth labels inconsistent	MAE: 0.18 (robust)
Equipment malfunction (sensor failure)	4.1%	0.45	Missing frames in sequence	MAE: 0.35 (degrades gracefully)
Overall	100%	0.245		0.245 (median)

**Table 14:** Ablation Study Results.

Configuration	mAP	Sub-Healthy AP	FPS	Robustness (@HIgh Turbidity)	Forecast MAE (4-Week)
Base:YOLOv13-L	49.5%	67.3%	95	43.7%	0.298 (ConbLSTM)
+WIO-Neck only	51.2%	69.5%	85	50.6%	0.298 (no improvement)
+CP-Head only	50.6%	71.4%	95	43.2%	0.298 (no improvement)
+Bio-Mamba only	49.7%	67.5%	91	44.1%	0.245
+WIO-Neck + CP-Head	52.4%	72.0%	87	50.2%	0.298
+WIO-Neck + Bio-Mamba	51.4%	69.8%	81	48.5%	0.233
+CP-Head + Bio-Mamba	50.8%	70.2%	88	43.4%	0.244
Full System	53.8%	72.8%	120	51.5%	0.245
Total improvement	+4.3%	+5.5%	+25 FPS	+7.8%	−17.8%

**Table 15:** WIO-Neck Hyperparameter Sensitivity.

DWT Wavelet	α (LL Enhance)	β (HH Suppress)	mAP	Robustness
Db4 (fixed α = 0.5)	0.3	0.7	51.8%	50.8%
Db4 (fixed α = 0.5)	0.5	0.5	52.1%	50.9%
Db4	0.5	0.7	53.2%	51.5%
Db4 (fixed α = 0.5)	0.7	0.7	53.2%	47.6%
Db4 (fixed α = 0.5)	0.5	0.9	50.4%	51.1%

**Table 16:** CP-Head Hyperparameter Sensitivity.

SupCon Temp (τ)	Loss Weight (λ_3_)	mAP	Sub-Healthy AP	Calibration Error
0.05	0.7	50.8%	71.8%	0.14
0.10	0.7	53.2%	72.8%	0.08
0.15	0.7	53.5%	71.9%	0.10
0.10	0.5	53.3%	71.1%	0.12
0.10	0.9	51.5%	72.1%	0.08

**Table 17:** Bio-Mamba Hyperparameter Sensitivity.

Hidden Dim (D)	Seq Length	Forecast MAE (4-Week)	FLOPs	Recommendation
128	672	0.268	52 G	Lightweight
256	672	0.245	72 G	**√**Optional
512	672	0.243	142 G	Overkill (diminishing returns)
256	336 (2-week)	0.198	48 G	Limited horizon
256	1344 (8-week)	0.289	135 G	Over-extension

**Table 18:** Cross-Site Generalization Performance. CR-Mix++ includes 8 distinct reef sites with varying baseline characteristics (depth, species composition, human disturbance). We trained on Sites A–G and tested on Site H to evaluate transfer learning capability.

Training Site (s)	Tested on Site H	mAP	Sub-Healthy AP	Robustness (@High Turbidity)
All sites (normal train–test split)	H (normal test split)	53.2%	72.5%	51.5%
A, B, C, D, E, F, G only	H (unseen)	48.2%	65.3%	46.8%
A, B, C, D, E, F, G, H (but different time periods)	H (future dates)	52.1%	70.8%	49.2%

**Table 19:** Per-Site Leave-One-Out Validation Results.

Left-Out Site	Mains Features	Depth	Dominant Species	mAP Drop
A	Branching coral, shallow	8 m	Acropora	−4.8%
B	Massive coral, medium	12 m	Porites	−5.9%
C	Bleached reef, deep	18 m	Mixed	−6.1%
D	Healthy reef, shallow	8 m	Acropora	−4.2%
E	Degraded reef, medium	13 m	Turf algae	−7.3%
F	Recovery zone, shallow	7 m	Juvenile coral	−8.1%
G	Deep reef, dark	22 m	Diverse	−6.5%
H	Mixed condition	14 m	Mixed	−5.6% (average)

**Table 20:** Per-Class Detection Confusion Matrix (Test Set). Rows = predicted class; Columns = ground truth class.

	True Healthy	True Sub-Healthy	True Bleached	True Dead
Pred Healthy	6680	485	28	11
Pred Sub-healthy	156	4380	287	64
Pred Bleached	42	198	5623	183
Pred Dead	18	69	262	6847

**Table 21:** Per-Class Recall and Error Patterns.

Class	Recall	Most Common Error	Error Rate
Healthy	89.1%	Missed (false negatives)	10.9%
Sub-healthy	82.6%	Misclassified as Bleached	17.4%
Bleached	90.4%	Misclassified as Dead	4.7%
Dead	95.8%	Misclassified as Bleached	3.8%

**Table 22:** Paired *t*-test Results for Per-Class Performance Improvements.

Comparison	Mean ΔmAP	Std Dev	t-Statistic	*p*-Value	Significant?
Healthy	+4.3%	1.2%	3.42	0.0006	✓✓✓
Sub-healthy	+5.2%	1.8%	6.50	<0.001	✓✓✓
Bleached	+3.0%	2.1%	3.21	0.0014	✓✓✓
Dead	+2.6%	1.5%	2.87	0.0042	✓✓✓

Notes: ✓✓✓ indicates statistical significance at the *p* < 0.01 level (two-tailed *t*-test).

**Table 23:** Training Efficiency.

Model	Training Time (300 Epochs)	GPU Memory	Convergence Epoch	Final mAP
YOLOv13 baseline	1.8 h	5.2 GB	75	49.5%
Coral-YOLO	2.2 h	5.8 GB	82	50.3%
Spectral-YOLOv13	4.2 h	7.5 GB	168	53.2%
Overhead vs. baseline	+2.4 h	+2.3 GB	+13 epochs	+3.7% mAP

**Table 24:** Real-Time Performance on Different Devices.

Device	FPS	Latency (ms)	Memory (GB)	Power (W)	Practical Deployment
RTX 4090 (NVIDIA Corporation, Santa Clara, CA, USA)	112	8.9	3.5	160	Lab/Ground station
Jetson AGX Orin (NVIDIA Corporation, Santa Clara, CA, USA)	45	22.2	3.8	25	AUV onboard ✓
Jetson Xavier NX (NVIDIA Corporation, Santa Clara, CA, USA)	12	83.3	4.2	10	Ultra-low power
CPU (Intel Corporation, Santa Clara, CA, USA, i9-13900K)	3	333	8.1	190	Not practical

Note: ✓ indicates the device is suitable for actual underwater AUV onboard deployment.

## Data Availability

The original contributions presented in this study are included in the article. Further inquiries can be directed to the corresponding author.
